# Training eye movements for visual search in individuals with macular degeneration

**DOI:** 10.1167/16.15.29

**Published:** 2016-12-27

**Authors:** Christian P. Janssen, Preeti Verghese

**Affiliations:** c.p.janssen@uu.nl www.cpjanssen.nl; preeti@ski.org http://www.ski.org/users/preeti-verghese; The Smith-Kettlewell Eye Research Institute, San Francisco, CA, USA; Department of Experimental Psychology and Helmholtz Institute, Utrecht University, Utrecht, the Netherlands; The Smith-Kettlewell Eye Research Institute, San Francisco, CA, USA

**Keywords:** *scotoma awareness*, *macular degeneration*, *preferred retinal locus*, *low vision*, *central field loss*

## Abstract

We report a method to train individuals with central field loss due to macular degeneration to improve the efficiency of visual search. Our method requires participants to make a same/different judgment on two simple silhouettes. One silhouette is presented in an area that falls within the binocular scotoma while they are fixating the center of the screen with their preferred retinal locus (PRL); the other silhouette is presented diametrically opposite within the intact visual field. Over the course of 480 trials (approximately 6 hr), we gradually reduced the amount of time that participants have to make a saccade and judge the similarity of stimuli. This requires that they direct their PRL first toward the stimulus that is initially hidden behind the scotoma. Results from nine participants show that all participants could complete the task faster with training without sacrificing accuracy on the same/different judgment task. Although a majority of participants were able to direct their PRL toward the initially hidden stimulus, the ability to do so varied between participants. Specifically, six of nine participants made faster saccades with training. A smaller set (four of nine) made accurate saccades inside or close to the target area and retained this strategy 2 to 3 months after training. Subjective reports suggest that training increased awareness of the scotoma location for some individuals. However, training did not transfer to a different visual search task. Nevertheless, our study suggests that increasing scotoma awareness and training participants to look toward their scotoma may help them acquire missing information.

## Introduction

One of the leading causes of vision loss that cannot be corrected optically or surgically is age-related macular degeneration (AMD; Friedman et al., 2004; Klein et al., 2011). AMD often results in vision loss around the fovea. In normal vision, the fovea provides high resolution and is used as the oculomotor reference for saccades. Individuals with binocular scotomas therefore need to find an alternate locus for fixation and as an oculomotor reference for eye movements. They tend to use a nondamaged location on the retina, often close to the boundary of the scotoma as an alternate fixation locus, referred to as the preferred retinal locus (PRL).

As the incidence of AMD increases with age, individuals who develop central field loss (CFL) due to AMD have the challenging task of adapting to a new PRL after decades of using the fovea as a fixation locus and oculomotor reference. Studies indicate that individuals adapt typically within 6 months to using an eccentric PRL for fixation (e.g., Chung, 2013; Crossland, Culham, Kabanarou, & Rubin, 2005), but they take much longer to use this locus as an oculomotor reference (White & Bedell, 1990). Indeed, efficient use of the PRL requires extensive training. Reading difficulties in individuals with vision loss have benefitted from extensive research (e.g., Chung, 2011; Fine & Peli, 1995a, 1995b; Fletcher, Schuchard, & Watson, 1999; Legge, Ross, Isenberg, & LaMay, 1992; Seiple, Grant, & Szlyk, 2011; Seiple, Szlyk, McMahon, Pulido, & Fishman, 2005).

Less focus has been on understanding how the PRL can be used efficiently in visual search settings. One of the few exceptions to this is a study by Kwon, Nandy, and Tjan (2013), who studied the development of a fixation locus/oculomotor reference in normally sighted participants with an artificial scotoma. They showed that visual search was a particularly effective method to train a fixation locus that developed later into an oculomotor reference.

The contribution of this paper is to describe a method that is aimed at helping individuals with CFL use their PRL more efficiently when searching for visual information. This complements other efforts that focus on helping individuals with AMD read better. Our rationale for training eye movements in individuals with CFL is based on the documented plasticity of the oculomotor system (as reviewed by Legge & Chung, 2016).

To illustrate the potential benefit of our proposed method, imagine a person with visual impairment due to CFL trying to find a can of beans in a cupboard. If the person has a large scotoma above the PRL in the visual field and directs fixation to the lower shelf of a cupboard, he or she might miss the can on the upper shelf if it falls into the scotoma. However, if the person is aware of the location of the scotoma and that it obscures visual information, he or she would look upward first and find the can of beans sooner. Indeed, search has been reported to be impaired for patients with AMD even in familiar search displays (Geringswald, Herbik, Hoffmann, & Pollmann, 2013).

The difficulty of visual search in individuals with CFL is exacerbated by the frequent lack of awareness of the scotoma as demonstrated by a recent study (Fletcher, Schuchard, & Renninger, 2012). In this study, 153 patients were asked whether they noticed any blind spots in their visual field during their first visit to an ophthalmologist specializing in low vision. About half of the patients did not notice anything. The other half noticed occasional vision problems but no explicit blind spots. In fact, only two patients visualized dark spots but under very specific circumstances (e.g., when waking up).

One hypothesis for why individuals with AMD might not be aware of the location of their scotoma is that perception compensates for the lack of input from the damaged retinal area (Komatsu, 2006; Zur & Ullman, 2003) by filling in the missing information. Initially, this information might not be accurate. Yet over the course of multiple saccades, the brain might integrate correct information from different parts of our visual field, including those that might initially be hidden by a scotoma.

However, information sampling may not be efficient in individuals with AMD, especially if they are unaware of locations where they are missing visual input. Furthermore, individuals with CFL typically make less directed eye movements that are of smaller amplitude compared to healthy adults (Renninger, Dang, Verghese, & Fletcher, 2008; Van der Stigchel et al., 2013).

The goal of our study is to train individuals with CFL to direct their current fixation toward the direction of their scotoma early in visual search to uncover hidden information. Ideally, such an eye movement is made with a single saccade. However, this might be challenging for individuals with extensive scotomas (e.g., 30° in one of our participants) as typical saccade amplitudes are less than 10° (Bahill, Adler, & Stark, 1975). Therefore, a realistic goal is to get participants to saccade in the direction of their scotoma.

The rationale is that saccades toward the scotoma will uncover information hidden by the scotoma and will make participants more aware of the relationship between their PRL and the scotoma. This work complements other recent studies that have focused on understanding and potentially improving functional vision and performance in a variety of everyday tasks, such as grasping, pointing, and smooth pursuit (e.g., Shanidze, Fusco, Potapchuk, Heinen, & Verghese, 2016; Sullivan & Walker, 2015; Verghese, Tyson, Ghahghaei, & Fletcher, 2016).

Nine individuals (four male, five female, age range 52–90, see Table 1) took part in our study. They were referred to us by Dr. Don Fletcher, an ophthalmologist specializing in low-vision rehabilitation. During a screening visit, we determined their ability to maintain fixation and measured the scotoma profile of each eye using a scanning laser ophthalmoscope (SLO). We also mapped out the binocular scotoma by asking individuals to detect images flashed at various locations on a tangent screen. Based on this assessment, we established a location in the visual field to train with the constraint that there was a total scotoma at this location and an unrestricted visual field in a location diametrically opposite.

**Table 1:**
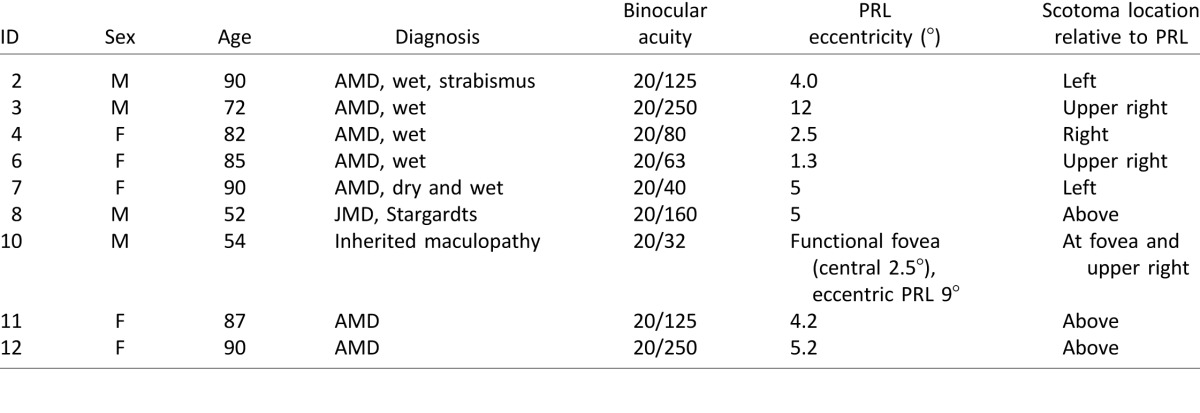
Participant information. *Notes*: Column 6 indicates the distance from the PRL to the foveal pit measured *monocularly* in the better eye. For participant 6, this does not correspond to the distance from the PRL to the center of the *binocular* scotoma because the monocular scotoma profiles are patchy with near foveal sparing.

**Figure 1:**
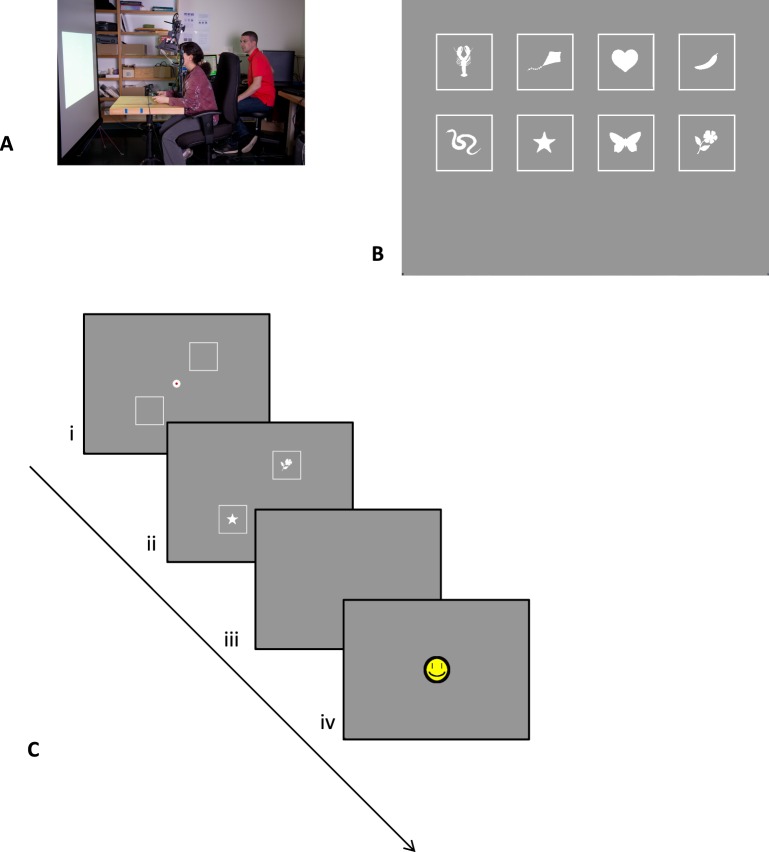
(A) Setup of the training task: Participants are presented with stimuli on a large projection screen. (B) Stimuli used in the same/different training task. (C) Timeline of the same/different training task: (i) Participant fixates a central marker with his or her PRL, and square outlines mark the locations of upcoming stimuli; (ii) stimuli appear when participant initiates trial and stay on for a limited time; (iii) participant indicates whether stimuli are the same or different; (iv) feedback is given.

During two visits in following weeks, we trained participants on a simple same/different task. In this task, which is similar to one used with healthy controls (Janssen & Verghese, 2015), participants had to judge whether two simple silhouettes were the same or different (see Figure 1 for procedure): one presented at the to-be-trained (scotoma) location and therefore not visible when fixating a central marker with the PRL and the other presented at a diametrically opposite location and clearly visible. The time available to scan the display was limited after the first eye movement. Thus, an efficient strategy was to move the eyes so that the stimulus behind the scotoma became visible. In early blocks of trials, the time available after the first eye movement was relatively generous (typically around 800 ms), and stimuli were presented at a fairly large scale (depending on scotoma size, between 3° and 8.75° wide). Over blocks of trials, the time available was shortened or stimulus size was reduced. In effect, this required participants to make more directed saccades toward the initially hidden location to be able to gather information there. By comparison, if saccades were directed to the opposite location where the stimulus was already visible, there might not be enough time to see both stimuli. The experimenter was present during the trials and reminded participants that one stimulus was hidden underneath their scotoma.

Based on the data, we address three research questions. Our first research question is whether training increases saccades toward the scotoma and thus improves the efficiency of information gathering. To this end, we analyze the fixation patterns (timing and location of fixations) during the training task and at the retention visit.

Our second research question is whether awareness of the scotoma also improved with training. To this end, we asked participants about their scotoma awareness during each visit in a semiformal interview. We also asked them whether they had experienced any benefit in their daily life.

Our third research question is whether performance transfers to other tasks. For objective measures of performance, we measured performance on the MNREAD task and a visual search task. Transfer was not expected on the MNREAD task as the nature of the task (reading fine print close to fixation) was very different from the task used during training (quickly finding information further away from fixation). However, it was an open question whether transfer would take place on a visual search task.

## Methods

### Participants

All our participants were patients of Dr. Don Fletcher, a local ophthalmologist, and had expressed their general interest to him in taking part in scientific studies. They were screened by him for general inclusion criteria (potential wet or dry AMD and binocular visual acuity of at least 20/400). Twelve participants expressed interest in participating.

Two participants failed to pass our additional screening tests (see Procedure). With one participant (participant 1), we were unable to calibrate the eye tracker for accurate measurement. In another participant (participant 5), the binocular overlap of the scotoma areas in the two eyes was minimal, resulting in the participant not missing any stimuli in our binocular scotoma mapping task (see Stimuli and materials). A third participant (participant 9) was excluded in our final analysis. We trained this participant based on an initial estimate of the scotoma. However, later analyses revealed that the participant did not have a stable PRL. Therefore, the location of the scotoma relative to fixation might have varied between trials and blocks, which makes results unreliable. Furthermore, this patient was diagnosed with advanced glaucoma soon after the retention visit, and we were unsure whether complications from glaucoma affected his visual field during the study.

Nine participants remained, and their characteristics are listed in Table 1. The diagnosis was provided by the ophthalmologist. In the lab, we measured binocular acuity using MNREAD^1^ (Legge, Ross, Luebker, & LaMay, 1989). PRL eccentricity and location were determined using microperimetry in an SLO as well as binocular scotoma mapping (described under Stimuli and materials) and input from the ophthalmologist.

The study was approved by the institutional review board at The Smith-Kettlewell Eye Research Institute and conformed with the Declaration of Helsinki. Informed consent was gathered as well as authorization for Dr. Fletcher to disclose personal health information to the research team.

### Design

Participants visited the lab for up to five visits (see Procedure). Visit 1 was a screening visit to determine whether they satisfied the criteria for inclusion and to map the binocular scotoma. During visits 2 and 3, participants were trained on a task (same/different task) that required uncovering a target initially hidden by the scotoma. Over blocks of trials, we gradually increased the difficulty of the task by decreasing the time that stimuli were visible after initiation of the initial saccade and/or reducing stimuli size. In effect, this required participants to make more directed eye movements in the direction of the scotoma. Our primary analyses focus on whether eye movements toward the scotoma were faster and more accurate after training compared to before training (research question 1). We also tested how well performance was retained after 3 and 6 months (visits 4 and 5). In addition, we asked participants about their scotoma awareness (research question 2) and measured potential transfer to a visual search task (research question 3).

### Stimuli and materials

#### Same/different training task

The same/different task was intended to train participants to make saccades toward the scotoma area and is illustrated in Figure 1C. The task had a similar design to our study with normally sighted control participants using artificial scotomas (Janssen & Verghese, 2015). In the version used here, two visual stimuli were presented diametrically opposite each other, equidistant from central fixation with one hidden behind the participant's scotoma and the other clearly visible. Participants had limited time to make eye movements and then had to judge whether the stimuli were the same or different.

The stimuli were based on eight hand-selected stimuli of the Snodgrass and Vanderwart (1980) data set: a heart, star, butterfly, flower, banana, kite, snake, and lobster (see Figure 1B). Each shape was transformed into a bright, filled silhouette on a gray background with a white square border surrounding it.

Each block had 48 trials. Within each set of 16 trials, all eight stimuli were shown once in a “same” configuration and once in a “different” configuration with order randomized. All stimuli occurred equally often.

The procedure of each trial was as follows: The fixation marker was a white annulus filled with a red disk presented at the center of a gray background (Figure 1C, step i). When participants were ready, they fixated the marker and initiated a trial by pressing a trigger on a game controller. A trial started only if the fixational PRL location fell within a square tolerance window around the center whose size was set depending on each participant's fixation stability. When a trial started, the red center turned gray (same as background), and the two squares with silhouettes appeared (Figure 1C, step ii). The location and size of the stimuli was determined by the experimenter such that one of the two stimuli fell approximately inside the scotoma of the participant while the other was clearly visible. To make this assessment, the experimenter used data from the binocular scotoma mapping task, microperimetry data from the SLO (both discussed later in this Materials section), and information from the clinician.

As soon as the participant's fixational PRL fell outside the tolerance window around fixation, a timer started. Stimuli were visible until the timer duration ended. Timer duration was set by the experimenter and decreased over blocks to encourage accurate saccades by the participant. A trial would also end if 2 s had passed since the trial start (e.g., when participants did not make an eye movement).

Finally, the participant had to indicate verbally whether they thought the two stimuli were the same or different (Figure 1C, step iii). The experimenter recorded this response with a keyboard. Participants were encouraged to guess if they were uncertain. Auditory and visual feedback indicated whether the answer was correct (cash register sound and smiley face) or incorrect (slamming door sound and grumpy face; Figure 1C, step iv).

As the task was challenging to the participants, the experimenter gave sufficient positive feedback to keep the participant motivated throughout the experiment. Although the focus of training was not to explicitly instruct participants where to look, the experimenter helped participants figure out the location of the hidden target, which, by design, was invisible when observers fixated the central marker. The trial started with the two square outlines that marked the stimulus locations (Figure 1C, step i), which could be visualized if the observer did not fixate the central marker. For the very first trial, the experimenter explicitly pointed this out to participants. As training progressed, participants learned the location of the hidden target as can be seen from the eye traces (e.g., Figure 5A).

The experimental software was developed in Python using the pylink 2.5 package. Stimuli were presented on a large projection (40.18° × 32.35°) at a distance of 90 cm. Each block started with calibration of the eye tracker (monocular tracking with an Eyelink 1000 eye tracker in tower mount setup and centroid mode; SR Research, Ottawa, Ontario, Canada). A 5-point calibration point was used for most participants as a 9-point calibration was hard to do. The eye tracker had a sampling frequency of 500 Hz. We used the default Eyelink criteria for saccade detection (minimum velocity of 30°/s; minimum acceleration of 8000°/s^2^).

The outcome metrics of this task were (a) percentage of correct trials, (b) distance of fixation from target, and (c) time point of fixation closest to target. Our hypothesis was that training would improve the accuracy of the saccades (landing more frequently close to the target) and the speed with which saccades in the direction of the scotoma were made.

#### One-target test task

**Figure 2:**
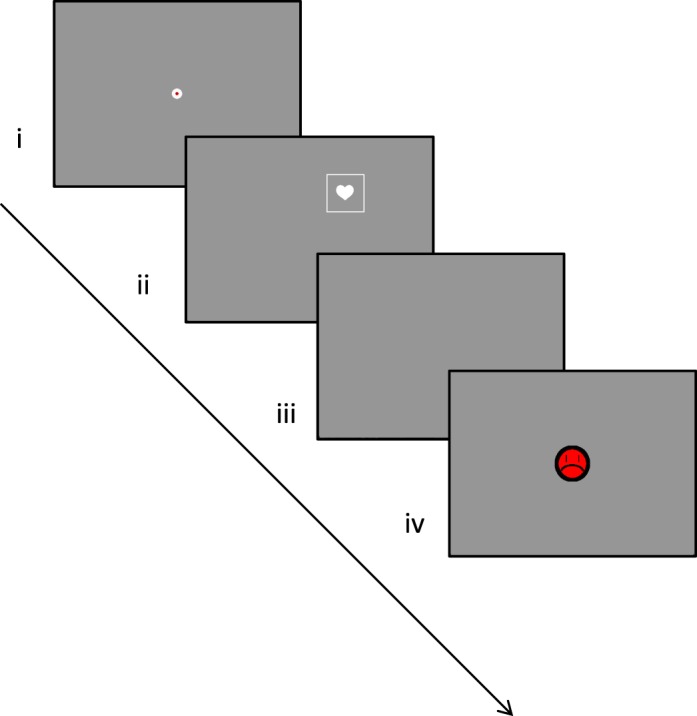
The one-target test task: (i) Participant fixates on central marker; (ii) after trial start, the stimulus appears at the scotoma location and stays on for a fixed time after the first saccade is initiated; (iii) participant indicates whether outline is filled or empty; (iv) feedback is given.

At the beginning of the retention visit (visit 4), we also used a variant of the same/different training task in which a single stimulus was shown at the location of the scotoma (see Figure 2). This task tested whether participants could make an effective saccade in the direction of the scotoma without a cue from the environment about its location (such as a visible stimulus on the opposite side). Each trial had a square outline in the scotoma that was either filled with a silhouette or empty. Participants were instructed that the stimulus would be presented in the location of the scotoma and were asked to respond whether a silhouette was present or absent. Each trial started with a red disk at the center of the screen (Figure 2, step i). When participants achieved fixation and indicated that they were ready with a button press, the stimulus was displayed at the scotoma location. The stimulus was then visible following a similar procedure as in the training phase (for a fixed time after a saccade was initiated) as illustrated in Figure 2, step ii. The screen was cleared, and participants had to verbally indicate whether a silhouette was present or absent (Figure 2, step iii). Participants then received visual and auditory feedback, similar to training (Figure 2, step iv). Outcome metrics were as before.

#### Optical coherence tomography (OCT)/SLO test

We assessed the monocular scotoma profile in each eye using the OCT/SLO (Optos, Marlborough, MA). Four tests were conducted monocularly, starting with the eye the participant deemed his or her “better” eye.

We used a line scan to take a two-dimensional, cross-sectional image of the retina. This was used to estimate the position of the biological fovea relative to the optic disk, which could be used later to estimate the eccentricity of the PRL from the fovea. A raster scan recorded a three-dimensional image of the retina to determine retinal thickness.

Microperimetry was used to map out the functional retina. Brief flashes (dots) were presented on the screen while participants fixated a cross. The experimenter selected test locations that were tested in random order. The central 25° of retina was tested with more focus on areas of interest, such as areas where there seemed to be a transition between affected and intact retina. After initial attempts to map contrast sensitivity at each location proved too long for participants, we set the flashes to maximum brightness to map out absolute scotomas.

Finally, we measured fixation stability. Participants were asked to fixate a cross without blinking for 10 s. The experimenter encouraged them verbally to stay fixated. We calculated the bivariate contour ellipse area (Steinman, 1965) based on the monocular data. In the main experiment, viewing was binocular although we tracked only the dominant eye. We checked whether participants were able to maintain fixation within a default tolerance window (given our knowledge of fixation stability). If participants were not able to maintain fixation within this window (a requirement for the trial to progress), we adjusted it.

#### Binocular scotoma mapping

Participants fixated on a central fixation marker while stimuli were flashed on a large (40.18° × 32.35°) screen to determine the location of the absolute scotoma. When participants indicated that they were ready for a trial with a button press, the trial was initiated if fixation was within the tolerance window. A white square outline filled with a white silhouette was presented for 200 ms at a randomly selected location (stimuli were taken from Janssen & Verghese, 2015). Participants were asked to verbally report whether or not they saw an object, and the experimenter entered their response on the keyboard. If participants saw only part of the stimulus, they were encouraged to indicate which parts were missing (e.g., “the bottom left corner”). We counted these trials as seeing part of the stimulus and scored them as 50% seen.

There were three versions of the experiment. In the first version, stimuli were 100 × 100 pixels (5° × 5°) and presented at 15 nonoverlapping fixed positions within a 500 (width) × 300 (height) pixel area centered around central fixation. Thirty trials contained a silhouette (each of the 15 locations was tested twice). Fifteen trials contained no silhouette and served as catch trials to check whether participants made up stimuli that were not there. In practice, this did not happen.

**Figure 3:**
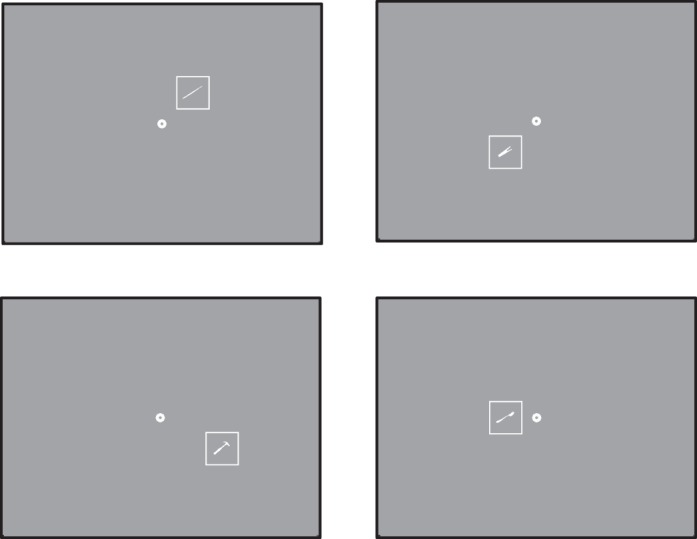
Example of four locations at which stimuli are presented in the binocular scotoma mapping task.

In the second version, stimuli were presented at a finer scale (50 × 50 pixels; 2.5° × 2.5°) at 35 nonoverlapping fixed positions within a 350 (width) × 250 (height) pixels area centered around central fixation. Seventy trials contained a silhouette (each location was tested twice). There were no catch trials in this setup as none of the participants had a false alarm on a catch trial in the first version. Figure 3 shows four examples of stimulus presentations in this version.

One participant (participant 10) had an intact fovea and a ring scotoma. To measure visibility for stimuli that were presented even further in periphery, we tested a third version in which the stimuli were 125 × 125 pixels (6.25° × 6.25°) and presented at 35 nonoverlapping fixed positions within a 750 (width) × 625 (height) pixels area centered around central fixation.

We monitored eye position at the start of the trial but did not check for eye movements during the 200-ms presentation, assuming that even if participants made an eye movement toward the stimulus during this short duration they would have seen some part of the stimulus. In effect, only stimuli that were truly not seen (even with a slight eye movement) are included. We consider this a conservative test of participants' binocular visual field.

Eight of the nine participants had a single, clearly developed peripheral PRL and a clear absolute scotoma. This was less clear for participant 10, who had a large central scotoma in one eye with an eccentric PRL and a similar scotoma in the other eye, albeit with foveal sparing. Binocularly, he routinely switched between the two PRLs but favored the foveal island. As the binocular scotoma was concentric to the fovea, we chose the training location based on his eccentric PRL, which put the scotoma in his upper right visual field.

#### Visual search task

To assess any potential transfer of training to other tasks, we used a visual search task in which participants had to report the number of blobs on an image of an indoor or outdoor scene. Participants completed 10 to 15 trials per session. Stimuli were shown on the projection screen at a distance of 90 cm.

**Figure 4:**
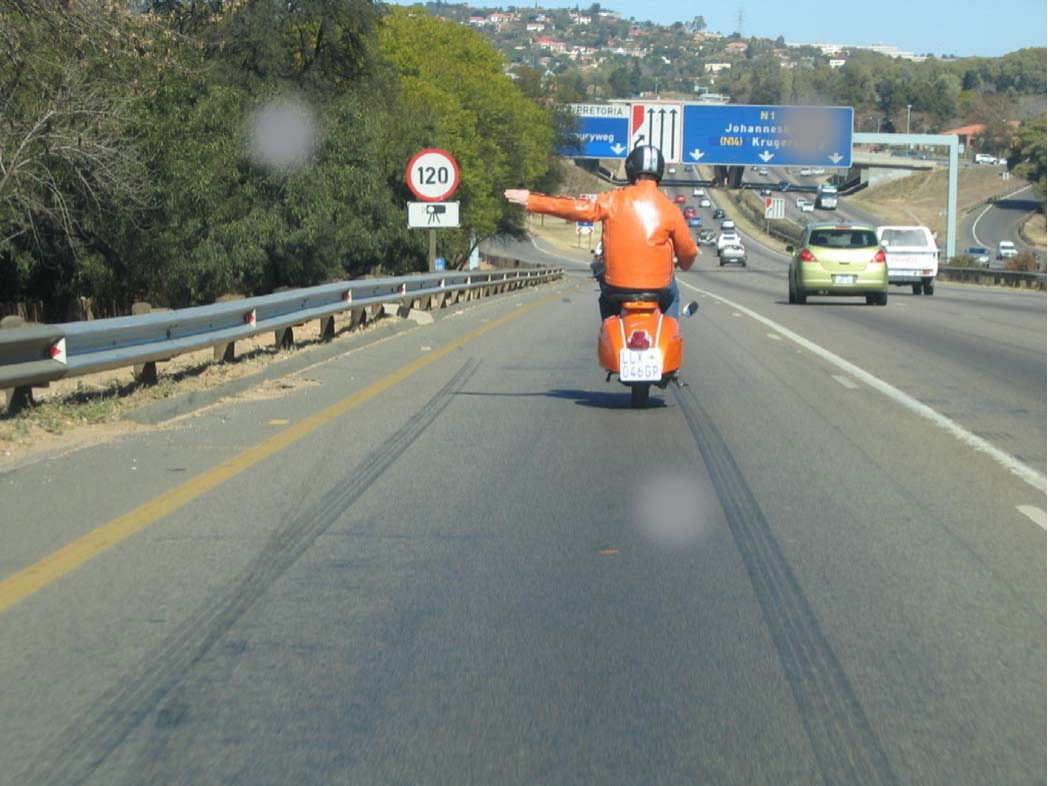
Example scene from the visual search task. Participants were asked to count all the blobs superimposed on an indoor or outdoor scene. Each image had between zero and nine Gaussian blobs, selected randomly.

In each trial, participants saw one scene (40.18° × 32.35°s), randomly selected from a database of 80 pictures of mostly outdoor scenes. Between zero and nine “blobs” (a two-dimensional Gaussian with a spatial standard deviation of 0.5°) were superimposed on random locations on a picture. The number of blobs was chosen randomly in each trial. Figure 4 shows an example.

Participants started by fixating with their PRL on a central fixation cross and indicating that they were ready for the trial to begin. The experimenter initiated the trial, which lasted for 10 s. At the end of the trial, participants verbally reported the number of blobs they had seen.

Two outcome metrics were used. First, we counted the number of blobs reported versus the number of blobs presented. Second, we counted the proportion of saccades made in the direction of the scotoma versus the opposite direction. Saccades (relative to last fixation) within an angle of 90° of the center of the target stimulus presented during training (i.e., inside scotoma) were classified as “toward” the scotoma. All others (also spanning a 180° angle) were classified as “away from” the scotoma. As the screen had a finite size, saccades could not go in one direction indefinitely. We therefore expected that training might result in more saccades toward the scotoma but not saccades in that direction alone.

#### Scotoma awareness questionnaire

At the start of each session, we asked participants about their subjective experience with their scotoma, following a predefined list of questions to document patients' scotoma awareness as well as any changes across sessions. Participants were asked to elaborate on their answers, and the initial question was used as a conversation opener. In two questions, we asked explicitly where participants thought they missed information in their visual field when they focused on a point: either a point on the wall or a point in the center of a clock (i.e., which digits might be unclear). Although we aimed to assess visibility relative to a PRL, some participants interpreted the questions as directing their old fovea to the point. This ambiguity limits the reliability of some results. The questionnaire can be found in the online supplementary materials.

#### Clock test

The clock test was an informal test administered to determine visual field deficits in addition to binocular scotoma mapping. The test was conducted in the first session and in subsequent sessions when answers to the questionnaire were unclear. The participant was seated in front of a large projection screen (40.18° × 32.35°) at a distance of 90 cm and shown a static image of a simple clock face. The clock had a central black circle, a black circular rim, and black ticks indicating the hours but no hands.

The participants were asked to maintain fixation on the clock center and if there were clock positions that were hard to see without moving the eyes. Then, the researcher listed all digits in random order and asked the participants how well they saw a particular digit while staying fixated at the center of the clock. The researcher sat diagonally from the participants to check that they did not move their eyes. Automated eye tracking was not used to ensure stable fixation. For participants, the clock test served as a way to directly test their subjective estimates of visibility on this test during the scotoma awareness questionnaire.

#### MNREAD task

The MNREAD task (Legge et al., 1989) measured binocular visual acuity. Participants were allowed to wear regular reading glasses but no magnifying glasses. The MNREAD chart was placed at a distance of approximately 40 cm and illuminated by an adjustable desk lamp. Participants were allowed to change the angle and brightness of the light (either off or in one of two brightness modes). The participants were instructed to read the sentences out loud, starting from the top. As per MNREAD instructions, participants were encouraged to guess a word that they had trouble reading even if the text did not make sense. The researcher noted the time required to read each sentence and the errors made.

#### Mini mental state exam

A mini mental state exam (MMSE) was conducted to assess cognitive impairment. Participant 1 did not perform the MMSE. All other participants passed the MMSE.

### Procedure

#### General overview

A summary of all tasks at each visit is given in Table 2. Participants came in for an initial screening visit. If they met the criteria for participation (see below), they were invited back for two more training visits that fell within a 1- to 2-week interval. The first retention visit occurred 2 to 3 months after the end of training. Five participants were able to come in for a second retention visit after an additional 2 to 3 months. During all visits, participants took breaks as frequently as they wished. This affected total duration of each session, which was between 2.5 and 3.5 hr. Below, we lay out the structure of each visit.

**Table 2:**
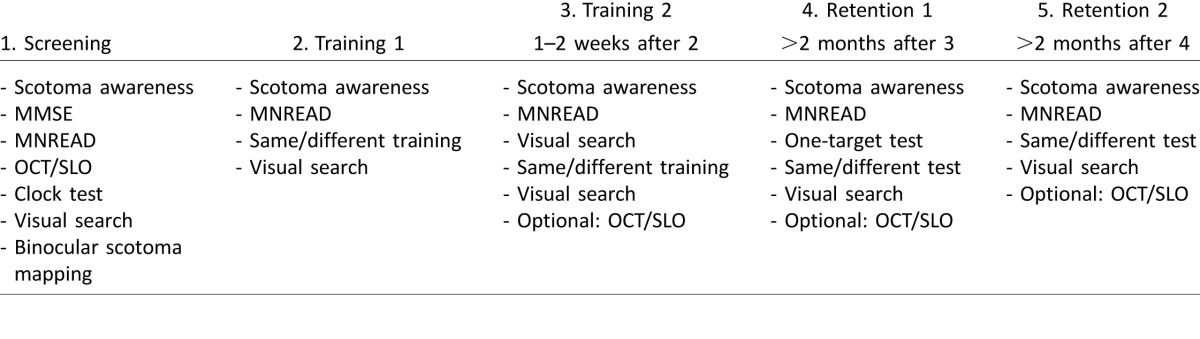
Overview of tasks performed during each visit.

#### Visit 1 (screening visit)

The goal of the first visit was to assess whether participants met the following criteria to take part in the study: no cognitive impairments (pass MMSE), binocular acuity of 20/400 or better, binocular scotoma, and a stable fixation with PRL that allowed calibration of the eye tracker.

Before arrival, participants received a brief description of the study by phone and received the consent form and protected health information form by mail. Upon arrival, the consent form and general structure of the study were discussed, and participants were given time to ask questions. After signing the forms, the experimenter administered the subjective scotoma awareness questionnaire, followed by the MMSE, and the binocular MNREAD test. These tests were followed by four monocular measurements on the OCT/SLO for each eye: a line scan, a 3-D topography, microperimetry, and fixation stability. The clock test was used to obtain a rough estimate of the binocular visual field and was followed by the binocular scotoma mapping test. This was followed by the visual search task.

#### Visits 2 and 3 (training visits)

The visit started with the subjective scotoma awareness interview and the binocular MNREAD test. This was followed by up to five blocks of the same/different training task (participants 7 and 12 occasionally stopped after three blocks as they were tired). The same duration and target size were used for the penultimate and last block of trials during visit 2. These same parameters were also used during the first block of visit 3. This allowed for multiple measurements under similar circumstances. Each visit ended with the visual search task.

During visit 3, the training task was also preceded by the visual search task. If a participant's OCT/SLO data was not complete during visit 1 and if the participant was willing, additional scotoma data were gathered during this visit.

#### Visits 4 and 5 (retention visits)

Retention visits occurred approximately 3 months after the end of training. During the retention visits, participants first performed the extended subjective scotoma awareness test and the binocular MNREAD test. During visit 4, participants performed one block of the one-target test task with parameter values set to their last training block on the same/different training task. During visits 4 and 5, participants performed two blocks of the same/different task again with the same parameters as during the end of training. Finally, participants performed the visual search task. If time permitted, we also collected OCT/SLO data to assess any changes in retinal characteristics. For one participant (participant 7), it was difficult to calibrate the eye tracker upon the first retention visit, so she only completed the one-target test, not the same/different training task.

## Results

### Research question 1: Does training direct saccades toward the scotoma?

#### Training parameters and performance

Table 3 provides an overview of the training parameters for participants at the end of the last training session. Throughout training, the parameters for the same/different task were adjusted to make the task more challenging, such that more directed saccades were necessary to uncover the target in the scotoma. At the end of the training, participants performed the task with stimuli that were visible for durations between 175 and 600 ms after the start of saccade. For comparison, these times ranged between 800 and 1000 ms at the beginning of the first training visit. For all participants, a reduction in time was possible. Similarly, the size of the target area was made smaller. At the start of the training, the targets had widths between 50 and 175 pixels (or 2.5° to 8.8°). At the end of training, this was brought down to widths between 50 and 120 pixels (or 2.5° to 6.0°).

**Table 3:**
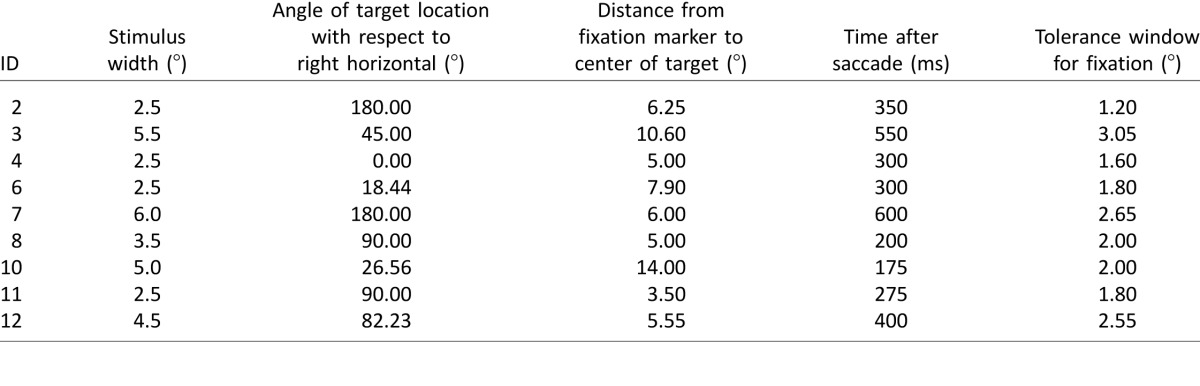
Experiment parameters at the end of training session.

Interestingly, the reduction in time and target size did not impair performance (see Table 4: percentage of correct responses on same/different judgment). A pairwise *t* test revealed no significant difference between performance at the end of visit 2 (first training session) and the end of visit 3 (last training session), *t*(8) = 0.730, *p* = 0.486. Similarly, there was no difference in performance between the end of training and performance on the one-target test task, *t*(8) = −0.361, *p* = 0.727. Finally, there was also no difference in performance between the end of training and performance on the same/different test at retention, *t*(7) = −1090, *p* = 0.312.

**Table 4:**
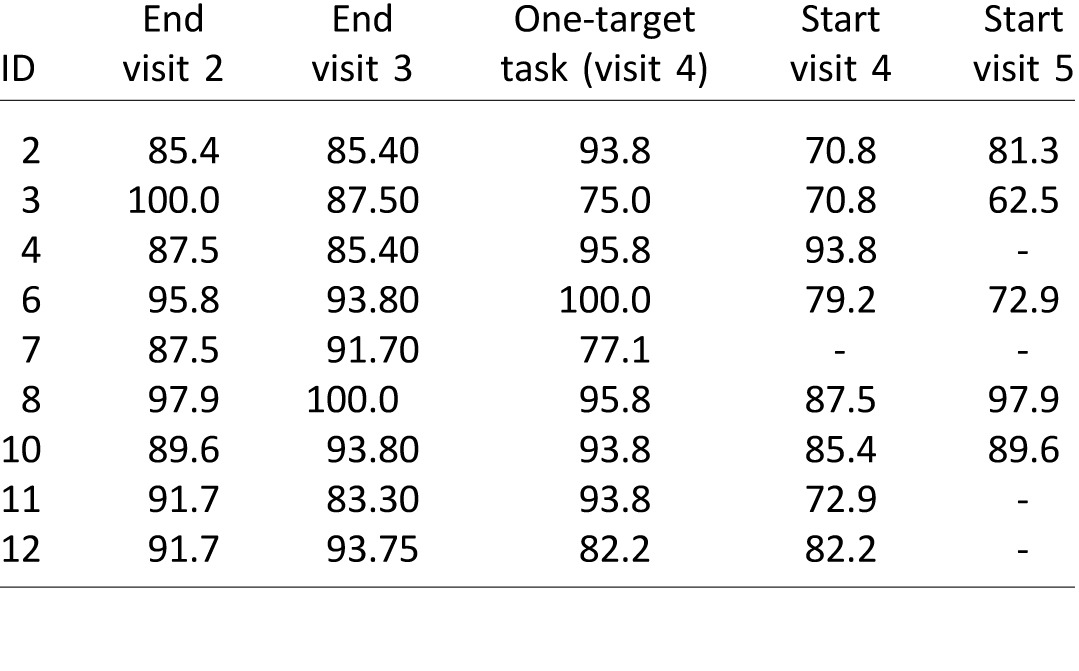
Percentage of correct responses on training task. *Notes*: Each column reports the performance of one block (48 trials) at either the end or start of a particular visit.

#### Eye movement pattern

Figure 5A, B,^2^ and C shows heat maps of fixations for three illustrative participants. Participants 8 and 12 performed well on the task; participant 6 had some challenges. Heat maps of all participants are included in the online supplementary materials.

**Figure 5A:**
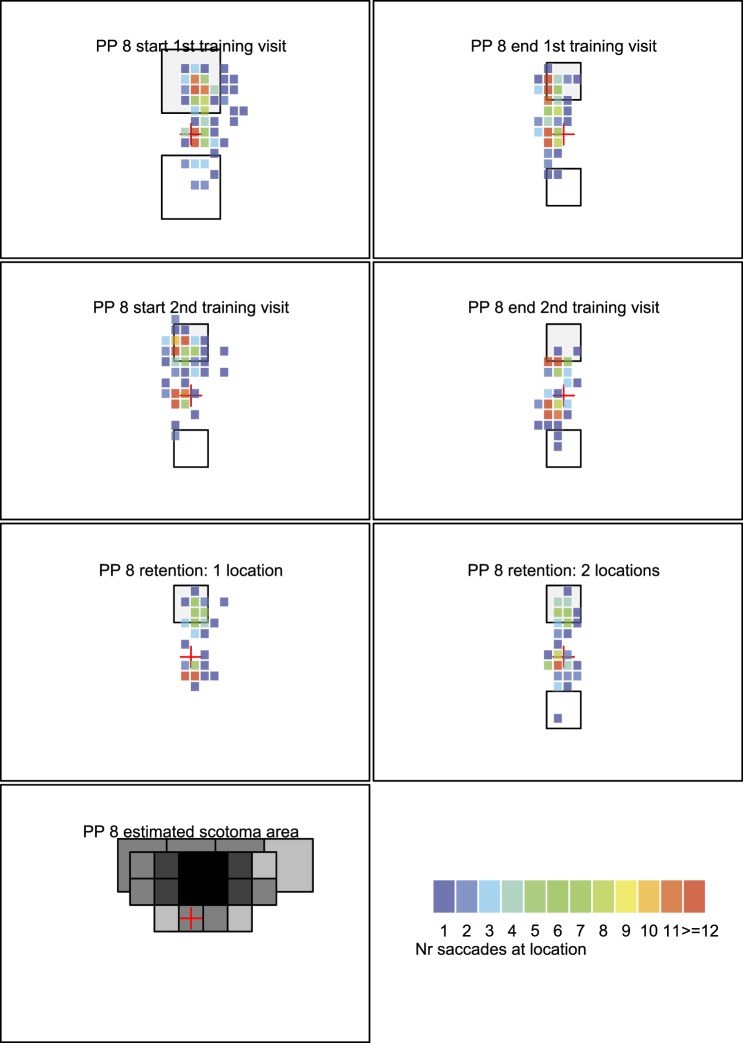
Heat map with fixation data from participant 8. Top row shows fixation locations on the first and last blocks of the first training session; the second row shows the first and last blocks of the second training session. The third row shows performance at retention for a block with only one stimulus (left) and with two stimuli (right). Bottom row shows the estimated binocular scotoma and the heat map legend showing the mapping of color to number of saccades.

**Figure 5B:**
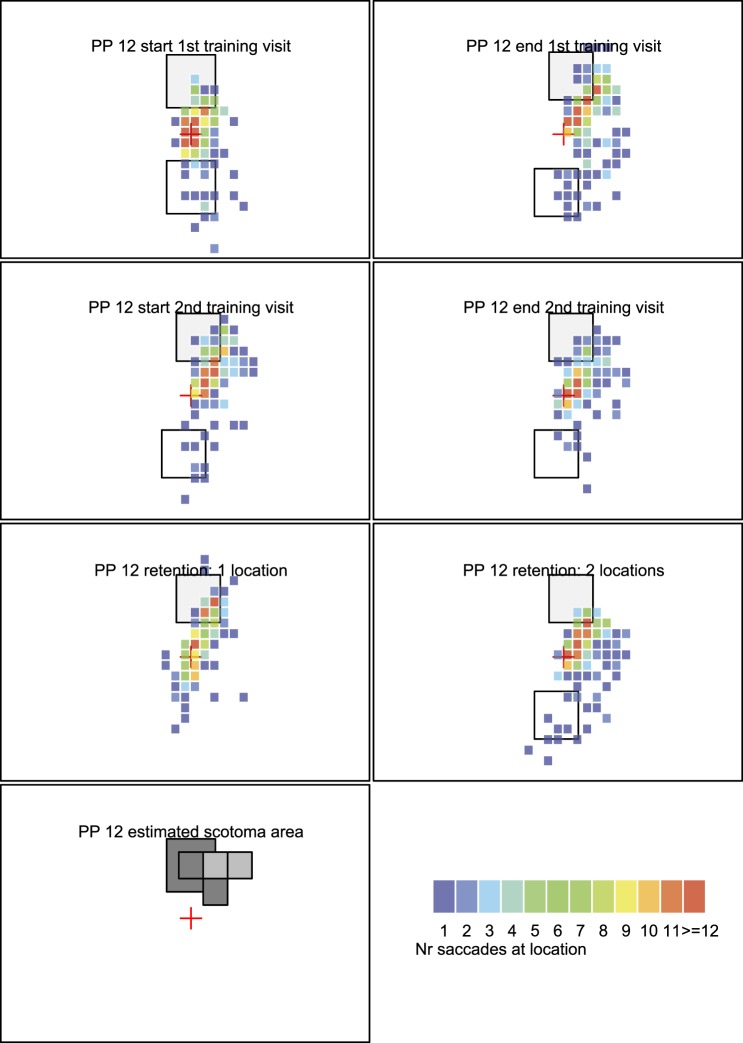
Heat map with fixation data from participant 12. Same organization of data as in panel A.

**Figure 5C:**
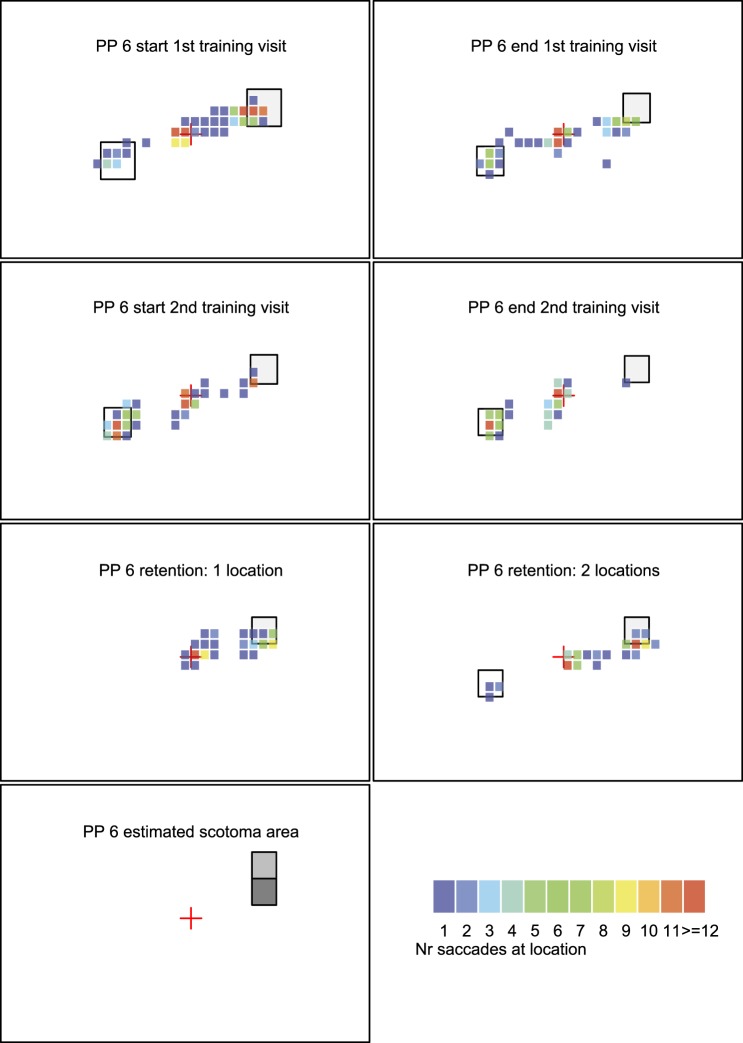
Heat map with fixation data from participant 6. Same organization of data as in panel A.

The bottom left figure in each participant's panel shows an estimate of the location of the binocular scotoma when the PRL is directed at the fixation marker as estimated on visit 1 in the binocular scotoma mapping task. The darker the square, the more frequently participants missed visual information in this area. A black square indicates that participants never saw a flashed target in this area, and a gray square indicates that participants either missed the target in some trials or indicated that they missed some information of the target (i.e., visibility counted as half).

**Figure 6:**
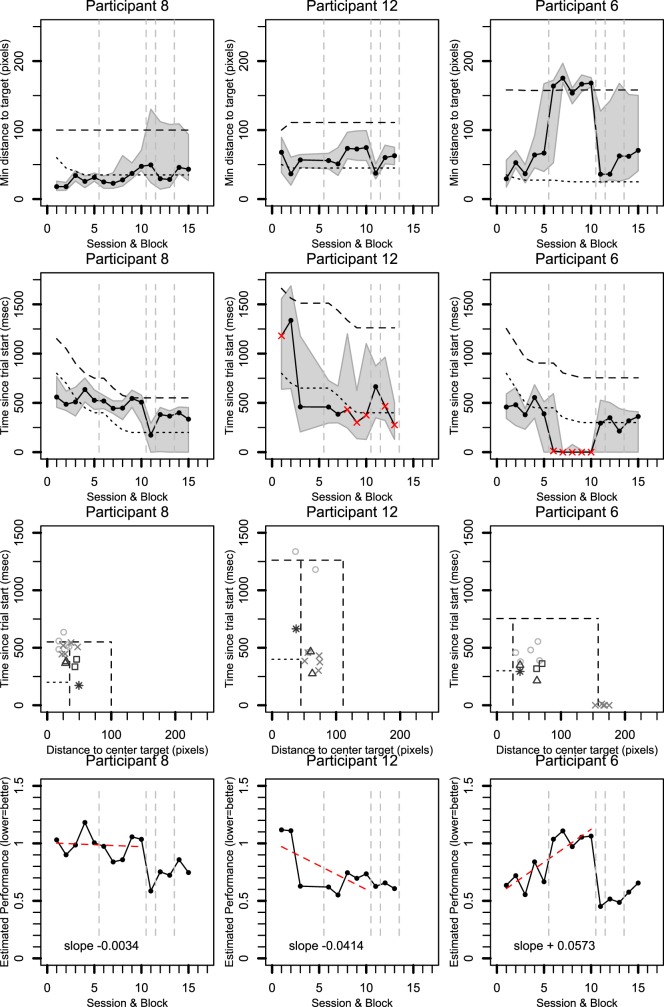
Saccade timing and fixation distance for three participants (columns). Rows 1, 2, and 4 show data over training blocks with visits separated by dashed gray lines. Solid lines show median data for each participant. Gray regions are bounded by the 25% and 75% quantiles. The first row shows the minimum distance between fixations and the center of the target. The second row shows when the fixation closest to the target occurred relative to trial start. Red crosses indicate trials in which fixation did not go toward the target. The third row combines the distance and time data for rows 1 and 2. The last row transforms the data in the third row into a normalized metric of distance from the origin. In all plots, lower values are better. See text for details.

In the heat maps, all three participants typically have a hot spot at or near the central fixation marker because this is where their fixational PRL was directed at the start of the trial. Participant 8 was able to direct his PRL toward the target in the scotoma (top of visual field, indicated with a light gray square) from the very first training block and continued to do so throughout training (Figure 5A, first two rows). At the retention visit, he directed his PRL toward the scotoma in both the one-target task and the same/different task although the number of fixations was slightly lower than during training (third row). Participant 12 was also able to direct her PRL toward the scotoma. This is visible in Figure 5B as each heat map has fixations in the target area (top of visual field). Moreover, with training, the number of fixations to the visible stimulus in the bottom half of the screen reduced. The participant also had many fixations in the target area after retention (third row in Figure 6) for both the one-stimulus (left) and two-stimulus versions (right) as indicated by the red squares in or close to the target area.

By contrast, participant 6 made many fixations in the target area (top right) during the first training visit (Figure 5C, top row). However, when the time deadline and the size of the stimulus got smaller at the end of the first training visit, she made fewer saccades toward the scotoma. In the second training visit with these stricter criteria, she consistently directed her PRL toward the distractor at the bottom left (second row). During training, this participant also remarked that she found the task of looking in the direction of her scotoma challenging. Surprisingly, during the retention visit, this participant was able to do the task successfully. The reason for the difficulty during training is unclear and might reflect a more reflexive tendency to look at the visible target when the time available for scanning was reduced. Our previous study showed that it took longer for normal controls to plan a saccade toward a target hidden by an artificial scotoma (Janssen & Verghese, 2015).

In general, six participants (participants 2, 3, 4, 8, 11, and 12) tended to direct their PRL mostly toward the scotoma area as indicated by their fixation patterns (see heat maps in online supplementary material). The remaining three participants (participants 6, 7, and 10) showed different results. In the following analyses, we investigate the timing and locations of saccade landing patterns more quantitatively.

#### Distance between center of target and closest fixation

Figure 6 shows data from the same three participants, one per column. The top row of Figure 6 shows the closest that participants' fixations got to the center of the target for each training block. The vertical axis shows the minimum absolute distance between PRL fixations and target center expressed in pixels (20 pixels is 1° visual angle). The horizontal axis shows the block number. Blocks 1 to 5 represent the first training visit (except for participant 12 who completed only three blocks), blocks 6 to 10 represent the second training visit, block 11 is the one-stimulus trial, blocks 12 and 13 are the first retention visit with two targets, and blocks 14 and 15 are the second retention visit with two targets. Dashed vertical gray lines separate the various visits.

Within each panel in the first row, the top dashed line reflects the distance of the target center from the central fixation marker. The lower dotted line indicates the distance of the target border from target center. Note that this distance becomes smaller over time for participants as the target size was reduced during the training. The dots (and solid line) show the median distance of the fixation closest to the target. The shaded gray region indicates the area between the 25% and 75% quantiles.

The participants who successfully directed their PRL to the target (participants 8 and 12) have a significant portion of their fixations (solid line, dots) inside or close to the target area (i.e., below or near the dotted line). For participant 8, there is more variation during the retention visit—indicated by the increase in the interquantile range (gray area).

Participant 6 (third column) makes many fixations inside or close to the target area during the first training visit and during the retention visit. However, during the second training visit (blocks 6 to 10), she does not get any closer than the central fixation marker (i.e., because she looked away from the target).

When we consider all participants (see online supplementary materials), four participants (participants 4, 8, 11, and 12) made fixations toward the target throughout the training. Four other participants (participants 2, 3, 6, and 7) have mixed results. For one participant (participant 10), performance seems relatively poor in that fixations are mostly away from the target.

#### Time at which fixations were closest to target

The second row in Figure 6 shows the time at which the fixations were closest to the target center relative to trial start. Participant data again shows the median (dots connected by solid line) with 25%–75% interquantile range. The dotted line shows the time available to complete eye movements after a participant's eye position exceeded the tolerance window around the central fixation marker (scan time parameter). Notice how this training parameter decreases over blocks. The black dashed line indicates the total time available to complete a saccade toward the target after trial start. This was estimated as the sum of median saccade latency and the scan time parameter. Median saccade latency was measured in block 11 (one-stimulus trial) as the time from the start of the trial to the point when fixation instability exceeded the tolerance window for fixation around the central fixation marker.

In general, shorter times for the eye to get in the vicinity of the target are good. However, a short interval could also emerge because a participant did not get any closer to the target than central fixation at trial start. We marked such data points with red crosses (points for which eye position did not cross the tolerance window around central fixation).

Results show that participants who successfully directed their PRL toward the scotoma (participants 8 and 12) reached the target area faster with training although occasionally there was high variance in fixation times (e.g., participant 12). In total, six participants showed a pattern that was relatively similar to this (participants 2, 3, 4, 8, 11, and 12; see online supplementary materials).

As noted before, participant 6 did not look at the target during the second training visit (i.e., red crosses in the figure) but did so in the first training visit and in retention visits. Participant 10 looked at the target in the first training session but not afterward. Participant 7 showed some initial improvement, but this did not persist during the second training visit (red crosses). This participant did not complete the same/different task during the retention visit.

#### Overall performance: Combining time and distance traveled

Although the preceding metrics consider the distance to the target and the corresponding time separately, improvements in eye movement efficiency could occur on both fronts: Saccades can be both fast and accurate. The third and fourth rows of Figure 6 therefore combine these metrics. For the third row, the vertical axis shows median time since trial start (i.e., dots from second row), and the horizontal axis shows median values for the closest distance to the target (i.e., dots from top row). The square areas are made by combining the points from the dotted and dashed lines of the top two rows of Figure 6. Ideal performance lies toward the bottom left corner: Here saccades are both fast and accurate (i.e., land inside the target area).

Data for each visit are represented with different symbols: open circle (first training visit), cross (second training visit), triangle (first retention visit), and squares (second retention visit). A heuristic to interpret the symbols: The more edges the symbol has, the later in training it occurred. Asterisks mark the one-target session.

The improvement of participants 8 and 12 is visible as performance gets closer to the origin with training (e.g., crosses are closer than circles) and remains good at retention (triangle and square). This is mostly because these participants made faster saccades later in training.

For participant 6, performance is good overall with improvement at retention (triangle, squares) compared to initial training visit (circles). However, during the second training session (crosses), performance is far from the origin because the participant did not look toward the scotoma.

To further quantify this combined time–distance metric, we expressed performance in each block using a normalized Euclidean distance (Figure 6, bottom row). For this metric, we normalized the time of closest fixation by the estimated total time available for the trial. Similarly, we normalized saccade distance by the distance between central fixation and center of the target. The dashed horizontal and vertical lines in the third row of Figure 6 represent normalized values of one. Using these normalized values, we calculated the Euclidean distance for each point relative to the origin. A lower value reflects better performance.

For the training trials (blocks 1 to 10), we also fit a linear trend line (red line) and calculated its slope. A negative slope suggests improvement over time; a positive slope suggests that performance got worse. A value close to zero suggests performance remained stable despite the fact that the task became harder with training.

Participant 12 shows clear improvement with training as indicated by the large negative slope of the trend line. For participant 8, improvement remained relatively stable with only a slight slope. This was also the case for three other participants (participants 2, 3, and 4; see online supplementary materials). For participant 11, there was a slight positive slope that was very close to zero (see online supplementary materials).

For participant 6, there is a positive slope: Performance got worse in the second training session. Two other participants also showed a slight positive slope (participants 7 and 10) but not as extreme as participant 6.

On the one-stimulus retention block (block 11), all participants except one (participant 7) have a similar or lower normalized Euclidean distance compared to their final training session. For the retention blocks with the same/different task (blocks 12 to 15), participants who had stable or improved performance over training either performed similarly to the end of training (participants 2, 3, and 12) or better (participant 8). For one participant (participant 4), it is unclear whether performance is similar or a little worse due to the variance in performance across the two retention blocks. In addition, participant 6 was better at the task.

To summarize, the Euclidean metric suggests that performance remained stable or improved over training for the majority of participants even though time and location constraints of the task became stricter. In addition, the majority of these participants managed to retain the performance at least 2 to 3 months after initial training.

### Research question 2: Does awareness of the scotoma improve with training?

The good retention of performance suggests that participants were able to act appropriately to compensate for missing information due to the scotoma. We examined whether this improved performance was accompanied by an increased awareness of the scotoma using data from the initial interviews at the start of each visit.

Over time, some participants explicitly confirmed their knowledge of the scotoma location. Most vocal was participant 3 (scotoma in top right), who reported that he noticed for the first time that the stock values were on the top right of the TV screen during the financial news. He also noticed that it became easier for him to find traffic signs. Participant 6 (scotoma in top right) made similar statements about daily tasks, such as noticing an exit sign in the top right. Participant 8 thought before the training that his scotoma was mostly in the top left of the visual field, but after training, he reported that he was aware he missed information in the upper visual field in general.

To quantify this further, we analyzed participants' responses during the scotoma awareness interview to the question asking where they missed information in their visual field relative to their PRL. We labeled their report as accurate if it was consistent with the objective assessment of their binocular scotoma location from mapping (the location we trained). We labeled their report incorrect if they indicated a location that did not have an absolute binocular scotoma. We labeled it as unclear if a damaged but untrained area was mentioned as this does not reflect learning during training. We also labeled an answer as unclear if participants replied that they missed information at center as this might, in hindsight, be due to the ambiguity of the questions. We intended to probe the location of their scotoma in the visual field with respect to their PRL whereas some participants responded about the location of the scotoma relative to their fovea.

The results do not show a consistent pattern. Three participants correctly identified their area upon their initial visit. For two (participants 3 and 10), this persisted, but for one (participant 7), the estimate was incorrect or unclear at later visits. One participant (participant 8) initially correctly noticed part of his scotoma but learned that the scotoma was wider during the training and correctly reported a larger area as of visit 3 and onward. Another participant (participant 12) reported the scotoma area correctly from the second visit onward, and another participant (participant 6) became aware of the scotoma location by the retention visit. The other three participants (participants 2, 4, and 11) consistently gave replies that were incorrect or unclear.

### Research question 3: Does performance transfer to a visual search task?

The scotoma awareness results are encouraging as they suggest that people knew more about their scotoma after the training. However, it relies on statements that were hard to verify (e.g., gaze in everyday life under different lighting circumstances). To assess transfer, we also looked at performance in a more natural visual search task (Figure 4).

#### Accuracy in reporting blobs

To start, we analyzed participants' accuracy at reporting the number of blobs present in each scene. Each image had from zero to nine blobs. We calculated the absolute difference between the number of blobs present and the number of blobs reported. A one-way ANOVA with three levels revealed that there was no significant difference, *F*(2, 16) = 1.44, *p* = 0.265, between the number reported on visit 1 (before training: *M* = 2.0 blobs, *SD* = 0.8 blobs), at the end of visit 3 (after training: *M* = 1.7 blobs, *SD* = 0.6 blobs), and at the first retention visit (*M* = 1.7 blobs, *SD* = 0.5 blobs).

#### Direction of saccades

We calculated the percentage of saccades made in the direction of the scotoma. To do this, we only considered saccades that traversed at least 1°. For these saccades, we calculated their angular direction relative to previous fixation. We categorized these directions in a binary fashion—saccades that went either toward or away from the scotoma (see Methods)—and calculated the median proportion of saccades in both directions for each session^3^ and the 25% and 75% quantiles. The data for each individual is plotted in the online supplementary materials. A dashed line indicates the 50% mark. Three participants clearly make more saccades toward the scotoma compared to away from it (participants 3, 8, and 11). For four other participants (participants 2, 4, 6, and 12), the median eye movements are toward the scotoma in the majority of blocks, but for some sessions, there is no clear preference for either direction due to overlap of the interquantile range with the dashed line. In general, there is no consistent effect of training experience on this metric. We therefore cannot conclude that there is clear evidence of transfer of training to another task.

## General discussion

We trained participants with vision loss due to a central scotoma to make more efficient saccades. In the training task, participants had to make a same/different judgment about two stimuli, one of which was presented within the scotoma relative to initial fixation. Participants were able to make correct judgments even when the task was made harder by limiting the time to make saccades and by reducing the size of the target. The efficiency of eye movements (research question 1) varied among individuals. The large majority of participants (six of nine participants) made faster saccades with training. A smaller set (four of nine) continued to make accurate saccades inside or close to the target area. The successful participants also retained this performance when they returned for a retention test 2–3 months after the last training session.

When probed about the awareness of the location of their scotoma (research question 2), some participants provided anecdotes that suggested their awareness improved. However, as a group, the results varied. This was mostly because our questions were ambiguous, which made the answers hard to interpret. We also tested whether efficient saccades transferred to a more naturalistic visual search task (research question 3). The distribution of saccade direction in the search task did not show a consistent improvement as a result of training. We therefore conclude that the training did not confer a clear benefit to scanning eye movements in a search task with only 6 hr of training.

In our sample, individuals who show clear training benefits typically have a dense scotoma in the upper visual field (participants 3, 6, 8, 11, and 12). In daily life, gaze is usually directed ahead and below as there are fewer ecological reasons to look up. So it is possible that for individuals with a scotoma in the upper visual field information in their scotoma was not routinely uncovered in typical scanning behavior (i.e., directed straight ahead and to the lower visual field). Furthermore, these participants have large scotomas, so the information that is obscured by the scotoma is not uncovered due to fixation instability. Thus, the benefit to these participants may have occurred because we trained them to direct their PRL to locations that they do not inspect routinely.

### Relationship to prior work

Previous studies have shown that people who have lost functional vision in their fovea due to a scotoma adapt to using a stable eccentric PRL within approximately 6 months (Crossland et al., 2005). However, the efficient use of this PRL as an oculomotor reference requires more extensive training (White & Bedell, 1990). A large majority of the training has focused on helping individuals with a scotoma read better (e.g., Chung, 2011; Nilsson, Frennesson, & Nilsson, 2003; Seiple et al., 2011; Seiple et al., 2005) or improve oculomotor behavior, such as fixation stability and eye movement control (Seiple et al., 2011; Tarita-Nistor, González, Markowitz, & Steinbach, 2009).

Such training methods typically take around 6 weeks and suggest that training methods that focus on general eye-movement control are particularly effective (Chung, 2011; Seiple et al., 2011). Our training regimen also focused on training specific eye movements (directed saccades toward the scotoma area). However, this was done in a shorter time interval (two training sessions within 2 weeks). For the majority of participants, this training length was beneficial. It is an open question whether those who did not learn to move their PRL toward the scotoma would have fared better with a longer training period. Even the training of the PRL/oculomotor reference in young, normally sighted controls with an artificial scotoma (Kwon et al., 2013) took a minimum of 10 hr of training before showing consistent results.

Our participants with CFL performed well when compared to control participants with an artificial scotoma. In a study by Janssen and Verghese (2015), healthy controls performed a similar same/different judgment task in which one stimulus was hidden by an artificial scotoma in the periphery. The participants were also under time pressure with only 200 to 300 ms to finish saccades. In that study, gaze patterns also varied among participants, but a majority of participants were able to make efficient saccades toward the hidden target. In trials in which initial saccades were toward the scotoma, the saccade was initiated later compared to trials in which the saccade was directed toward the visible stimulus in the location opposite the scotoma. For participants with CFL in the current study, saccades that were aimed toward the scotoma typically had a latency of several hundred milliseconds (with medians roughly around 300 to 700 ms). Some participants in the current study made saccades that landed close to the target area within a similar time frame. With training, the saccades were directed more frequently toward the scotoma, and latency is close to that of controls (e.g., compare current latency results with distributions in figures 2 and 4 of Janssen & Verghese, 2015).

### Limitations and future work

We made a detailed study of performance during and after training participants to look toward their scotoma. The number of participants in our study is small but is comparable to some other studies with extensive measurements over multiple sessions (e.g., Chung, 2013; Seiple et al., 2005; Shanidze et al., 2016; Verghese et al., 2016).

In the training paradigm, we only trained participants for 10 blocks of 48 trials each over a period of 2 weeks. Those who were able to direct their PRL toward the scotoma maintained their performance upon a retention visit 2 months later. However, some participants did not consistently look toward the scotoma. As successful reading training studies train for around 6 weeks (e.g., Chung, 2011; Seiple et al., 2011), one potential avenue to explore is whether participants' performance would improve more consistently if training were longer.

Participants did not show clear signs of transfer to a visual search task. Future work should therefore look into whether transfer can be demonstrated with a different choice of task or with more training. Specifically, previous work has suggested that reading can benefit from training individuals with CFL to make horizontal eye movements with the PRL (Seiple et al., 2011). Future work is required to determine whether the oculomotor training method we used has benefits for reading and whether it can specifically address the clustering of fixations that is correlated with slow reading in individuals with CFL (Calabrèse, Bernard, Faure, Hoffart, & Castet, 2016).

Some participants gave clear indications of having more awareness about their scotoma after the training whereas for others this was unclear (mostly due to ambiguity of the interview question). Future work should explore what aspects of the training or visit helped improve awareness and how this can be improved with others.

We measured fixation stability monocularly. Most patients had at least one eye with good fixation stability and were able to maintain fixation in the binocular display, suggesting a relatively stable binocular PRL for fixation. However, it is possible that they used a different fixation locus during the task as we know that some individuals with CFL use multiple PRLs (Whittaker, Budd, & Cummings, 1988). For instance, in another task in our lab, participant 10 switched fixation between a foveal island and an eccentric PRL at about 10°, such that the large central scotoma was in the upper right visual field. We expected that he might use his eccentric PRL at least part of the time and placed the target in the scotoma in the upper right visual field. However, he seemed to consistently use the foveal island for fixation in this task, so given his ring scotoma, he was missing information in a large annulus around fixation, not just in the upper right location. This might explain why the training task did not increase the proportion of eye movement to the upper right.

Yet another limitation of our study is that participants were only allowed to move their eyes to compensate for their scotoma. This was due to our use of a headrest to allow for better eye tracking. However, in everyday life, there are other ways in which people might compensate for vision loss. For example, they could move their head, use information from other sensory modalities (e.g., audio), or use accessibility technology to gather additional information about their surroundings.

Participants in our study were required to move their eyes quickly to unveil the target before the timer ran out. In that sense, our task is somewhat artificial. However, there is potential learning associated with uncovering a hidden target. In a recent study, normally sighted participants were asked to identify noisy targets among distractors in the periphery (Verghese & Ghahghaei, 2013). When participants received immediate feedback that removed the noise and disambiguated target from the distractor, they quickly learned to make efficient eye movements during active search. This suggests that the immediate feedback our participants received when they uncovered a hidden target might itself have been an important mechanism for helping individuals make more efficient saccades. Future work can therefore explicitly investigate whether time pressure is needed to improve saccade efficiency.

Finally, although our study was aimed at training individuals to make saccades toward the scotoma, it is possible that other eye movement strategies could have been used in our task. For the particular case of two stimuli that are diametrically opposite, participants could have made a saccade in a direction orthogonal to the axis of the two stimuli, thus potentially uncovering both simultaneously. Our goal was to train a strategy that would have a more general application in the real world, i.e., to uncover information hidden by the scotoma in a rich visual scene.

## Conclusion

We introduced a novel paradigm for training saccade efficiency in individuals who have a binocular scotoma due to AMD. Two thirds of the participants appeared to benefit from training although the training effects did not clearly transfer to other tasks with active eye movements with only 6 hr of training. These results provide crucial information for further studies in this area to help individuals with vision loss due to AMD improve functional vision in tasks of daily living.

## Supplementary Material



Supplement 1

## Footnotes

1 We routinely use the MNREAD chart to test acuity in individuals with CFL because, in addition to acuity, it provides a measure of reading speed and a pattern of reading errors that helps determine the locus of the scotoma relative to the PRL.

2 The stimulus location shifts slightly from the second block onward. This is by design as the scotoma of this participant was slightly more toward the right than at top center. The participant could see the stimulus in the first training block (directly above central fixation) without an eye movement.

3 For the third visit, we only used data from the visual search test administered at the end of the training session.
