# Supplementary figures and images for: Training eye movements for visual search in individuals with macular degeneration
Source: J Vis. 2016 Dec 27;16(15):29. doi: 10.1167/16.15.29 (PMC5214619; doi:10.1167/16.15.29)

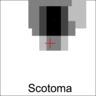

Supplement: Supplementary file 1 [file JOV-05446-2016-s02-ICON.gif]
