# Supplementary material for: Training eye movements for visual search in individuals with macular degeneration
Source: J Vis. 2016 Dec 27;16(15):29. doi: 10.1167/16.15.29 (PMC5214619; doi:10.1167/16.15.29)
Supplement: Supplement 1 [file JOV-05446-2016-s01.pdf]

Supplementary Material to:

Training eye movements for visual search in individuals with macular degeneration

Janssen & Verghese

Journal of Vision

This supplementary material contains the following files:

1. Heatmaps for participants 2, 3, 4, 6, 7, 8, 10, 11, 12. Layout of the heatmaps is similar to Figure 5 in the main manuscript.
2. Minimum distance to target plot for participants 2, 3, 4, 6, 7, 8, 10, 11, 12 (each in an individual plot). Layout is similar to the top-row of Figure 6 in the main manuscript.
3. Time since trial start plot for participants 2, 3, 4, 6, 7, 8, 10, 11, 12 (each in an individual plot). Layout is similar to the 2nd row of Figure 6 in the main manuscript.
4. Combined plot of time since trial start (vertical) and distance to target plot for participants 2, 3, 4, 6, 7, 8, 10, 11, 12 (each in an individual plot). Layout is similar to the 3rd row of Figure 6 in the main manuscript.
5. Estimated performance plot of time and distance for participants 2, 3, 4, 6, 7, 8, 10, 11, 12 (each in an individual plot). Layout is similar to the 4th row of Figure 6 in the main manuscript.
6. Proportion of saccades towards the scotoma in the visual search task for participants 2, 3, 4, 6, 7, 8, 10, 11, 12 (each in an individual plot). Horizontal axis plots the visit. (see text for detail)
7. Subjective questionnaire

1. **Heatmaps for participants 2, 3, 4, 6, 7, 8, 10, 11, 12. Layout of the heatmaps is similar to Figure 5 in the main manuscript.**

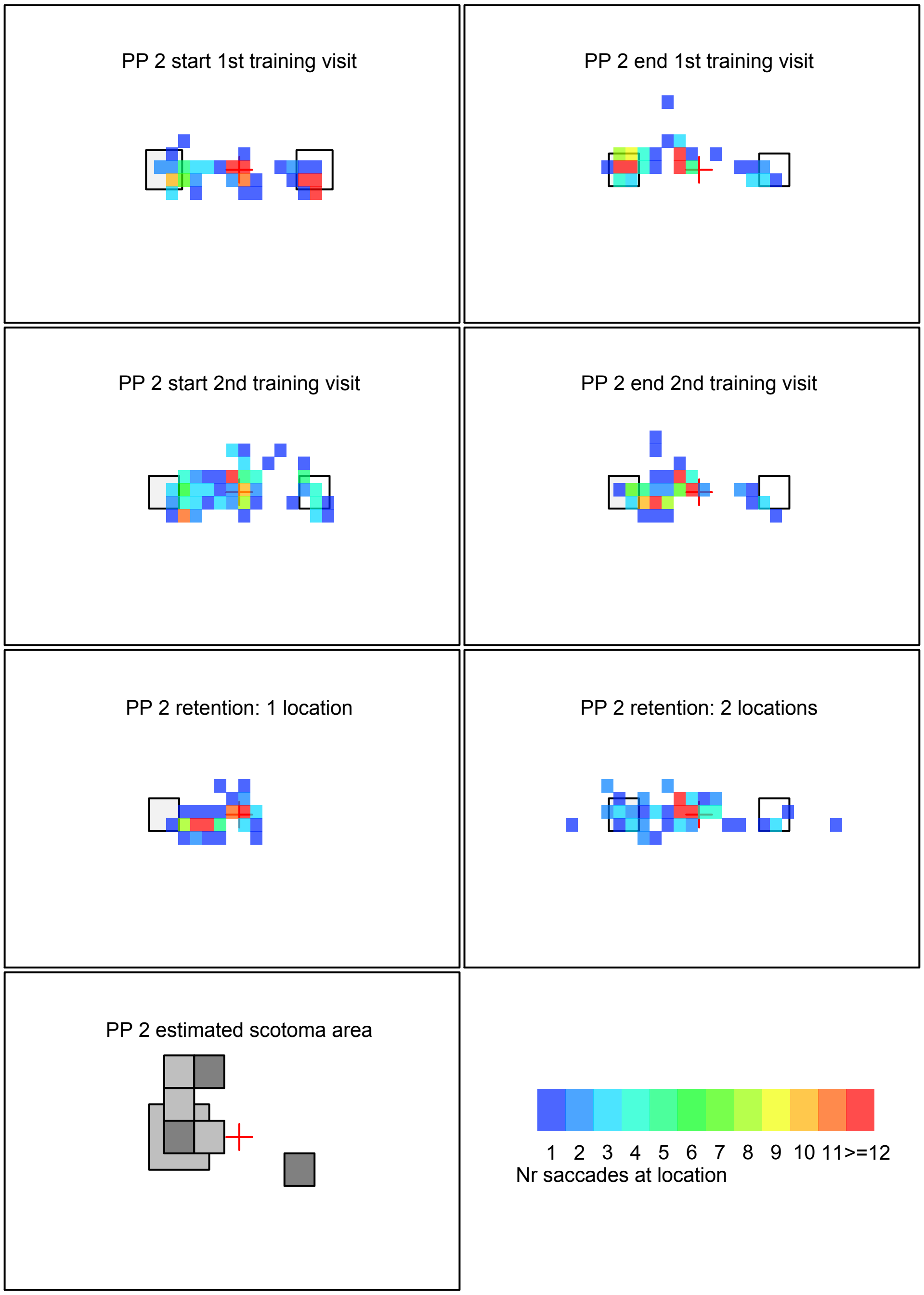

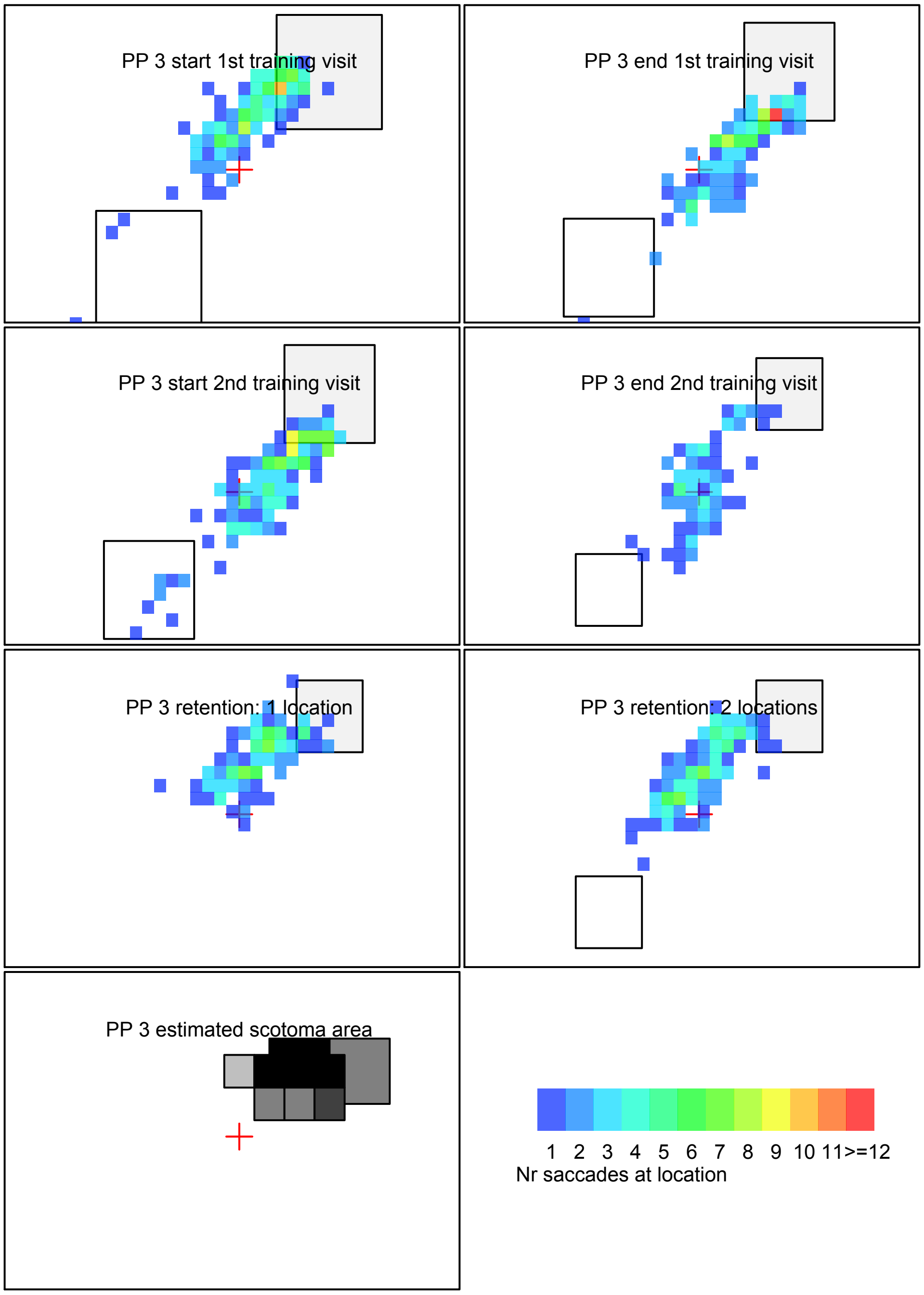
PP 3 start 1st training visit
PP 3 end 1st training visit
PP 3 start 2nd training visit
PP 3 end 2nd training visit
PP 3 retention: 1 location
PP 3 retention: 2 locations
PP 3 estimated scotoma area
1 2 3 4 5 6 7 8 9 10 11>=12
Nr saccades at location

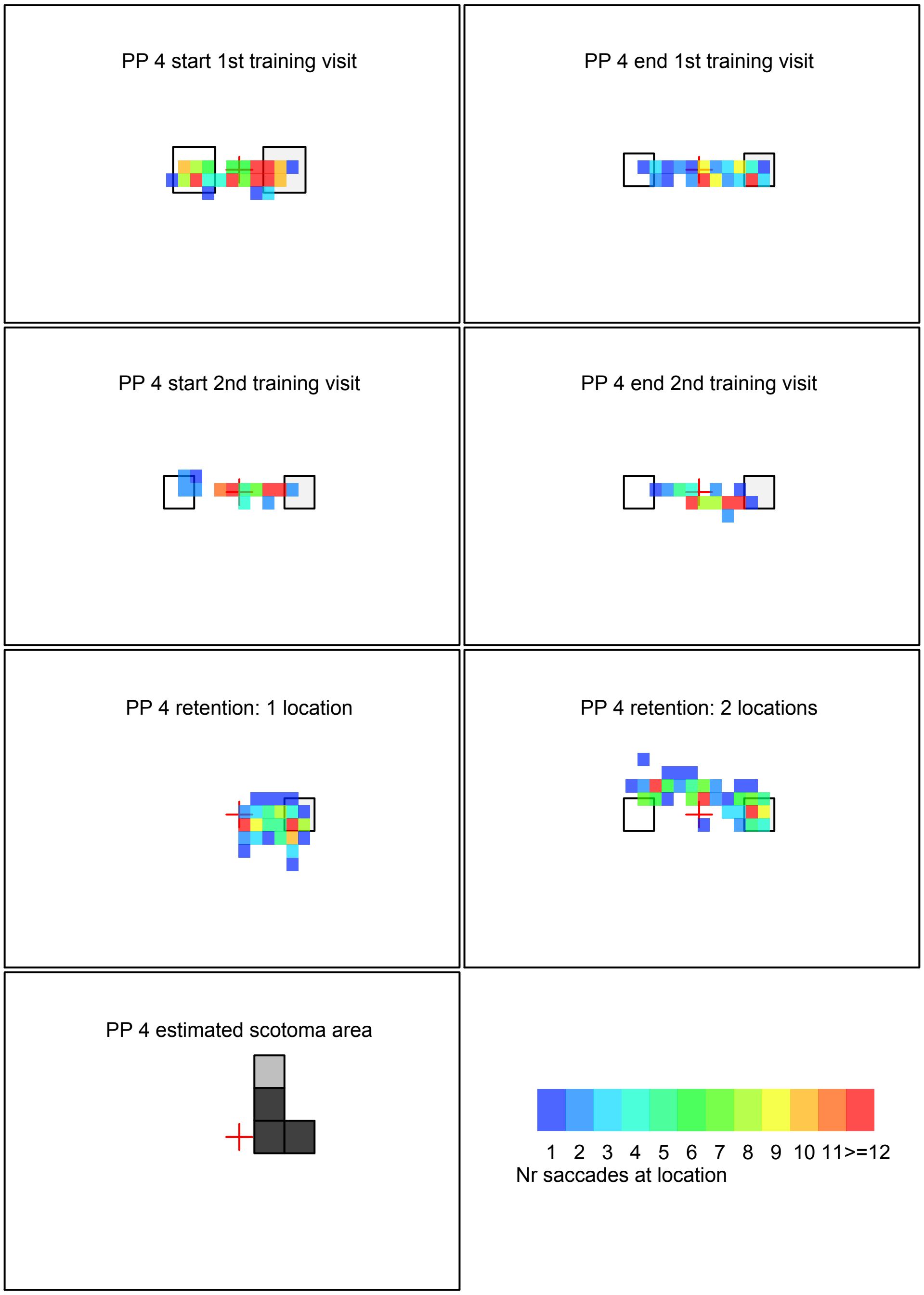
PP 4 start 1st training visit
PP 4 end 1st training visit
PP 4 start 2nd training visit
PP 4 end 2nd training visit
PP 4 retention: 1 location
PP 4 retention: 2 locations
PP 4 estimated scotoma area
1 2 3 4 5 6 7 8 9 10 11>=12
Nr saccades at location

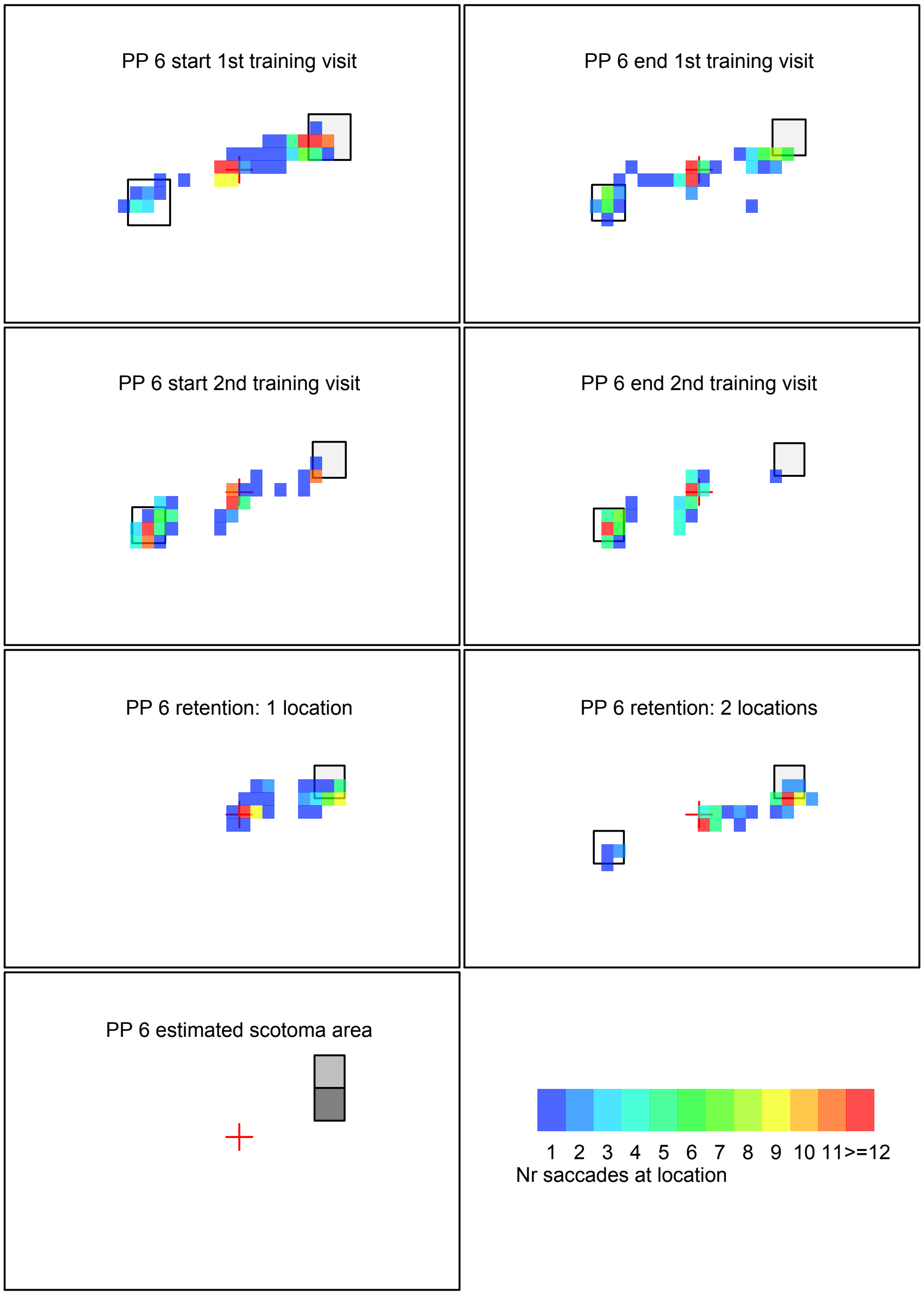

PP 6 start 1st training visit
PP 6 end 1st training visit
PP 6 start 2nd training visit
PP 6 end 2nd training visit
PP 6 retention: 1 location
PP 6 retention: 2 locations
PP 6 estimated scotoma area
1 2 3 4 5 6 7 8 9 10 11 >=12
Nr saccades at location

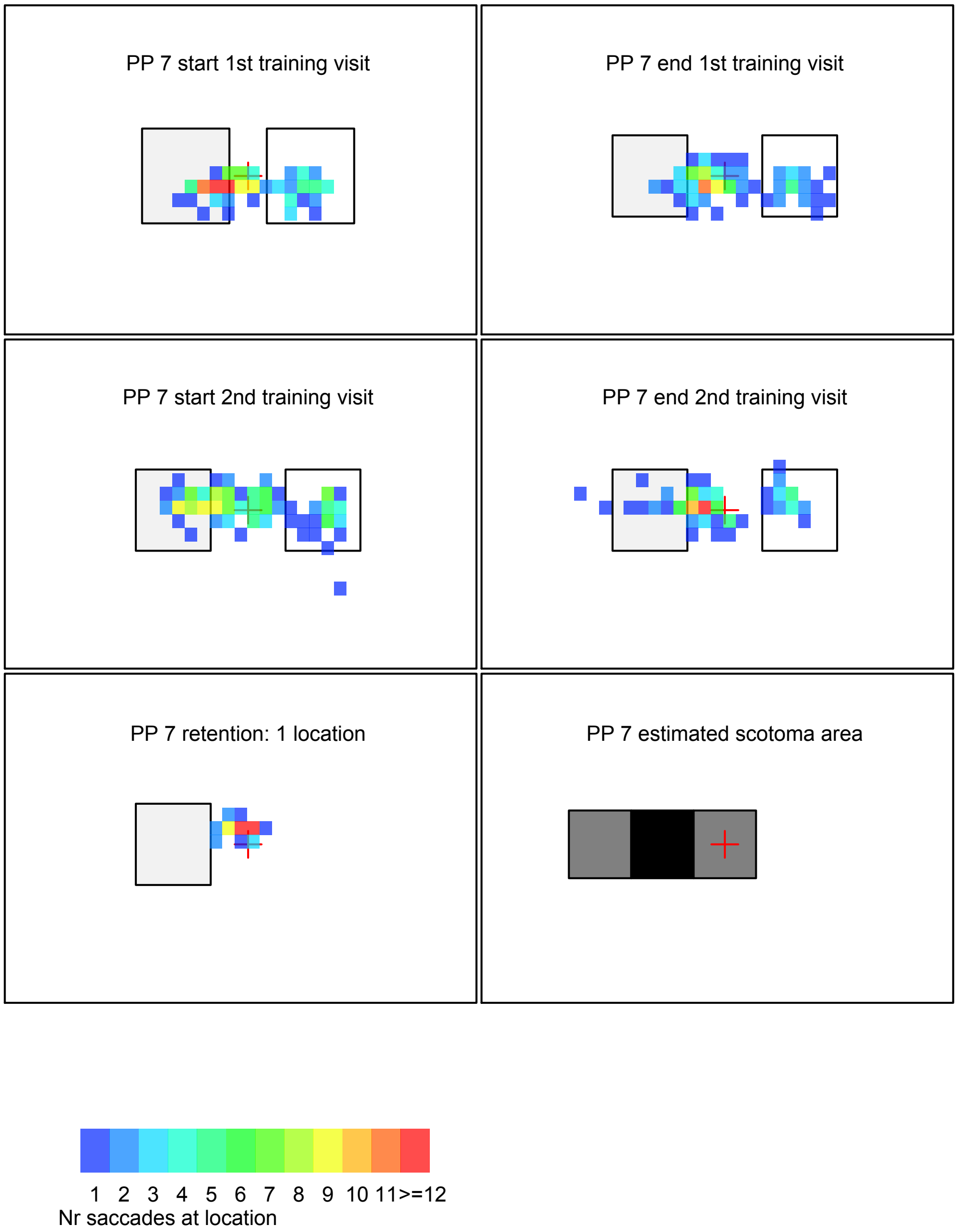
PP 7 start 1st training visit
PP 7 end 1st training visit
PP 7 start 2nd training visit
PP 7 end 2nd training visit
PP 7 retention: 1 location
PP 7 estimated scotoma area
1 2 3 4 5 6 7 8 9 10 11>=12
Nr saccades at location

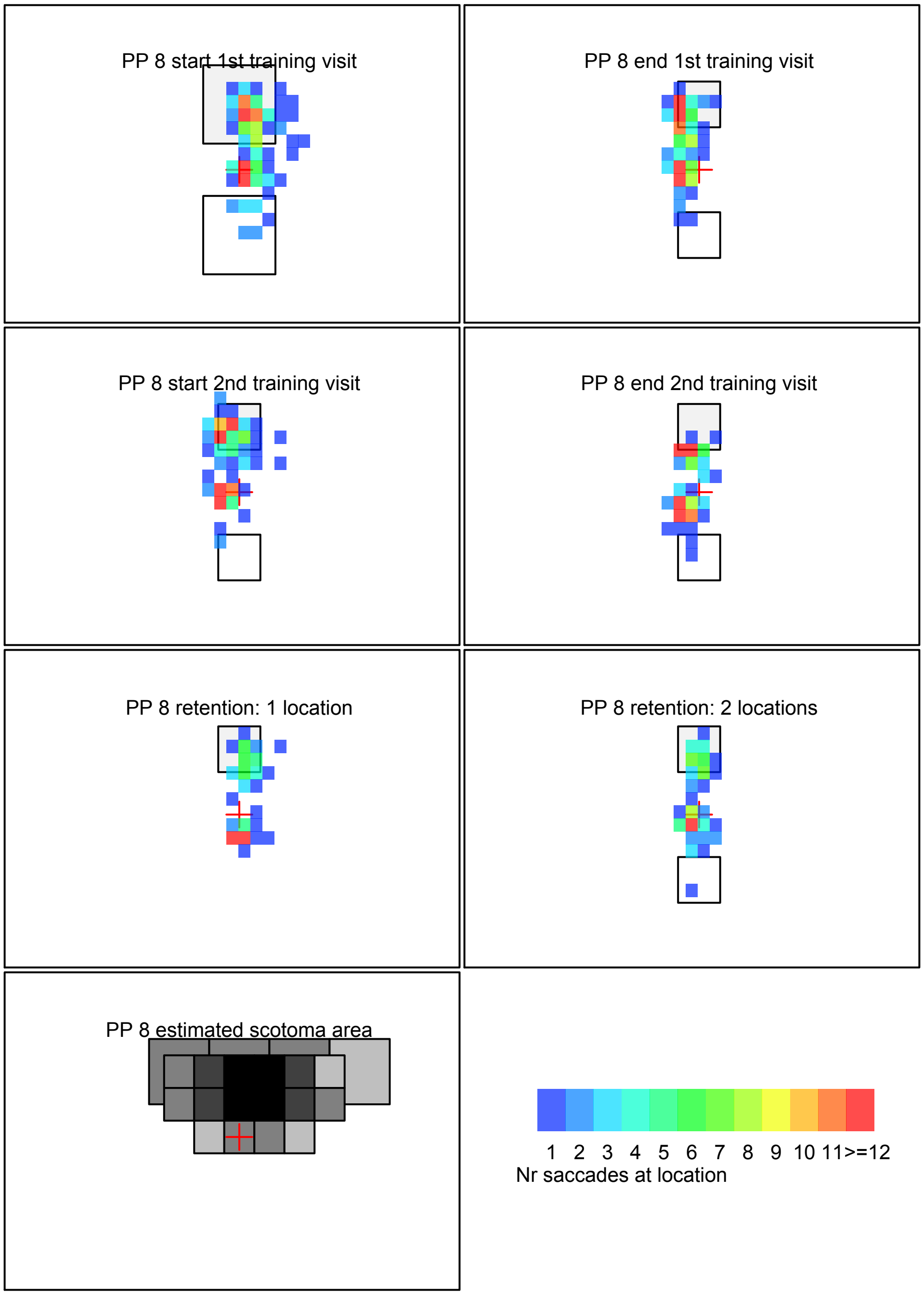
PP 8 start 1st training visit
PP 8 end 1st training visit
PP 8 start 2nd training visit
PP 8 end 2nd training visit
PP 8 retention: 1 location
PP 8 retention: 2 locations
PP 8 estimated scotoma area
1 2 3 4 5 6 7 8 9 10 11>=12
Nr saccades at location

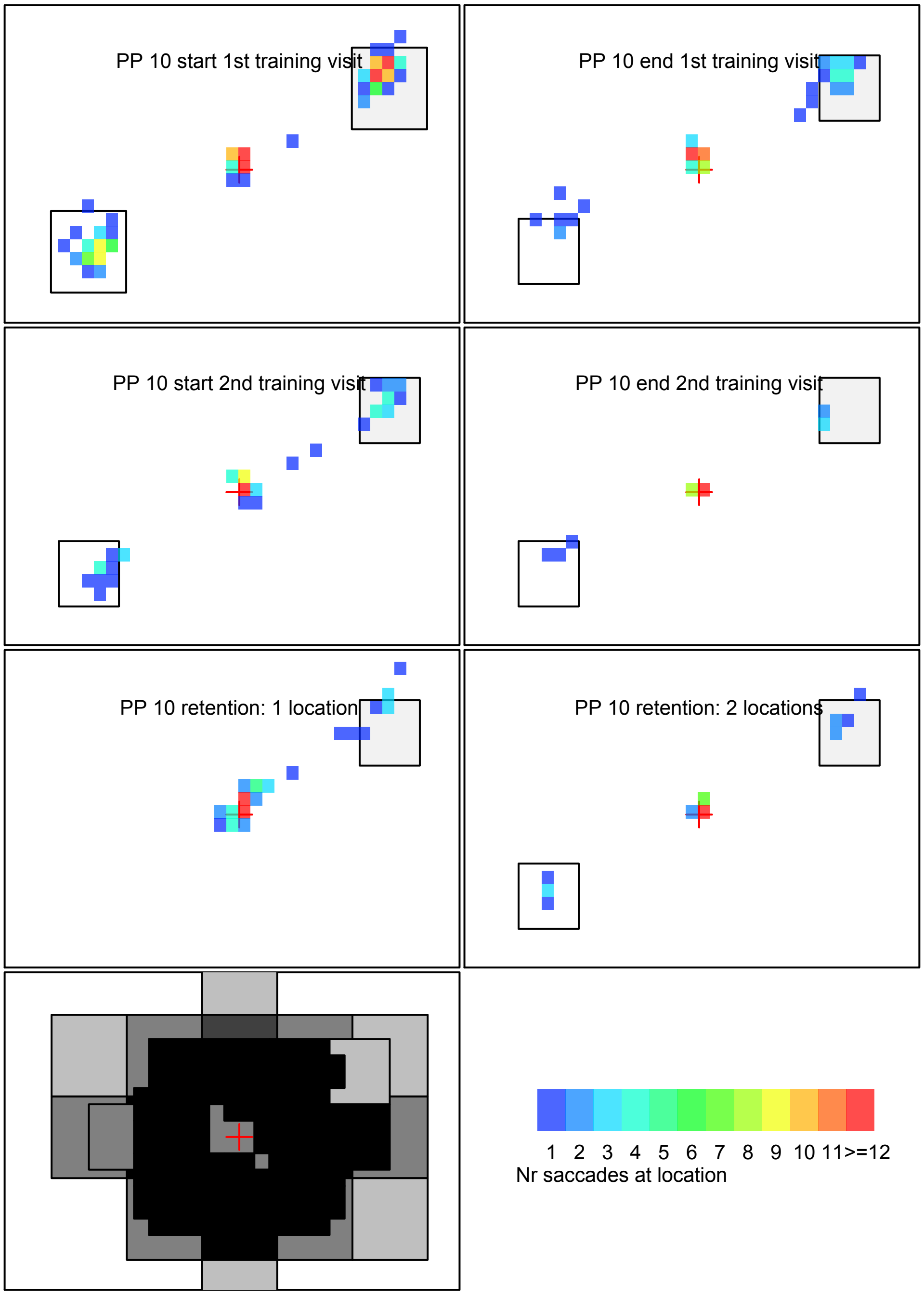
PP 10 start 1st training visit
PP 10 end 1st training visit
PP 10 start 2nd training visit
PP 10 end 2nd training visit
PP 10 retention: 1 location
PP 10 retention: 2 locations
1 2 3 4 5 6 7 8 9 10 11>=12
Nr saccades at location

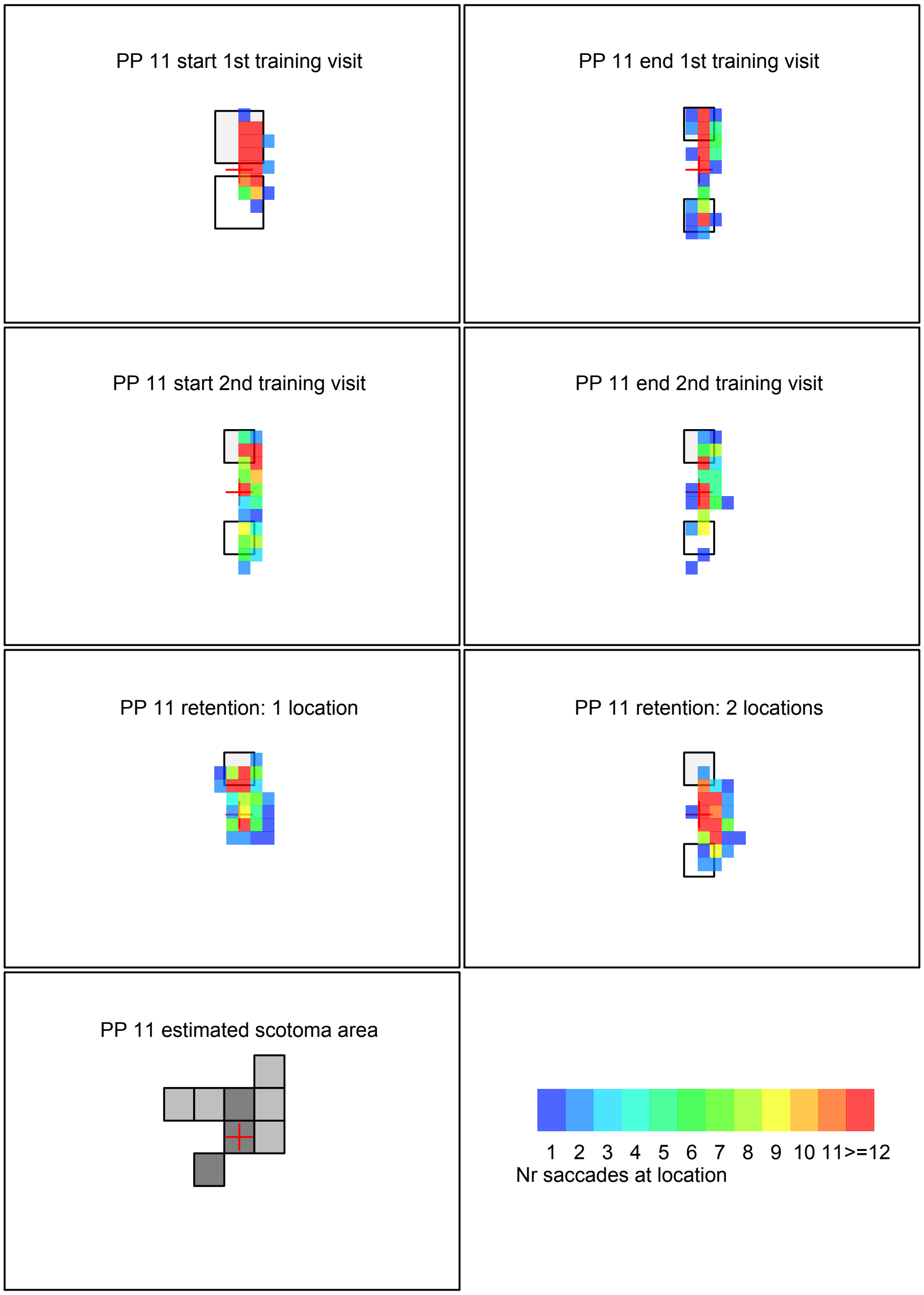
PP 11 start 1st training visit
PP 11 end 1st training visit
PP 11 start 2nd training visit
PP 11 end 2nd training visit
PP 11 retention: 1 location
PP 11 retention: 2 locations
PP 11 estimated scotoma area
1 2 3 4 5 6 7 8 9 10 11>=12
Nr saccades at location

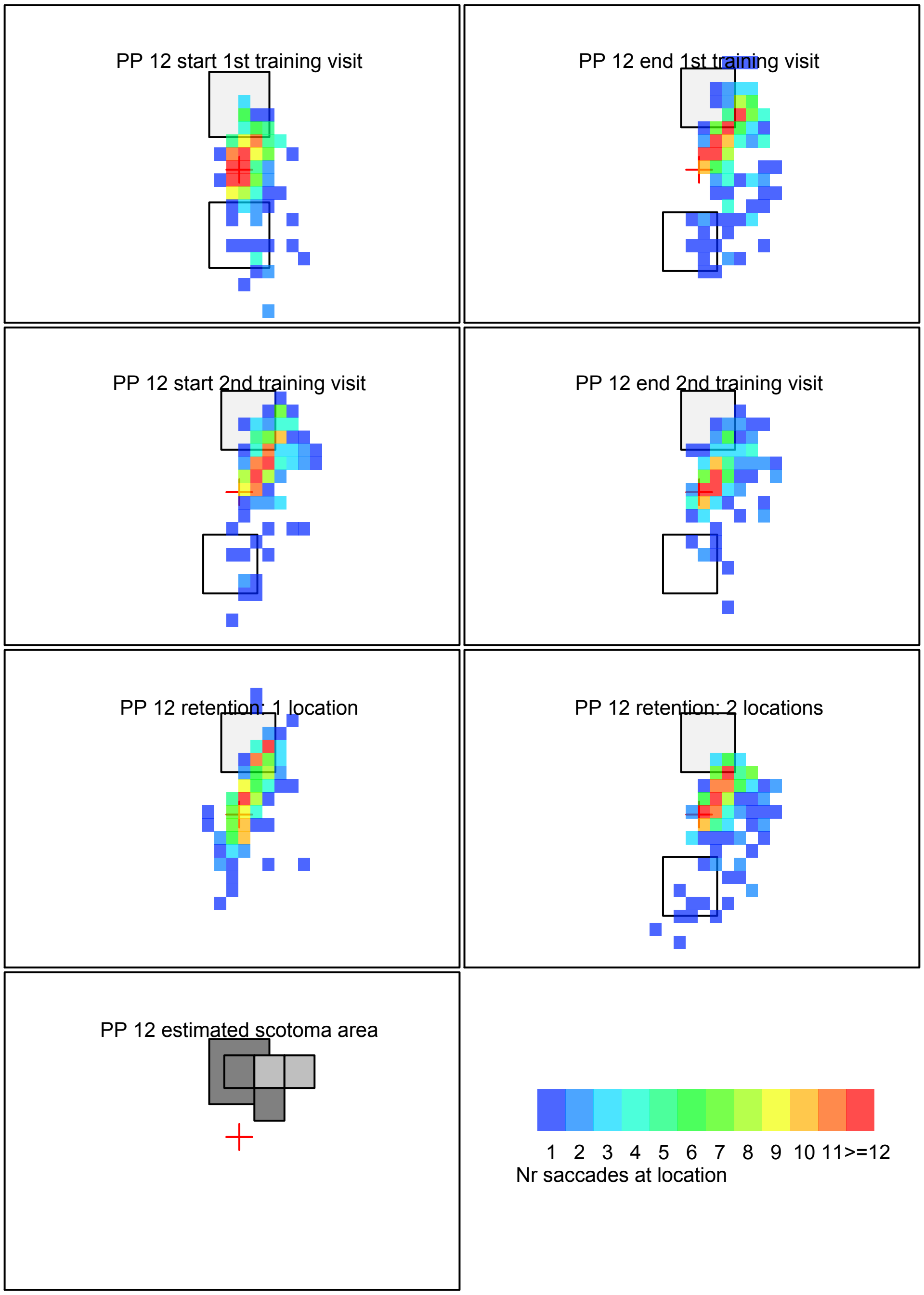
PP 12 start 1st training visit
PP 12 end 1st training visit
PP 12 start 2nd training visit
PP 12 end 2nd training visit
PP 12 retention: 1 location
PP 12 retention: 2 locations
PP 12 estimated scotoma area
1 2 3 4 5 6 7 8 9 10 11>=12
Nr saccades at location

2. **Minimum distance to target plot for participants 2, 3, 4, 6, 7, 8, 10, 11, 12 (each in an individual plot). Layout is similar to the top-row of Figure 6 in the main manuscript.**

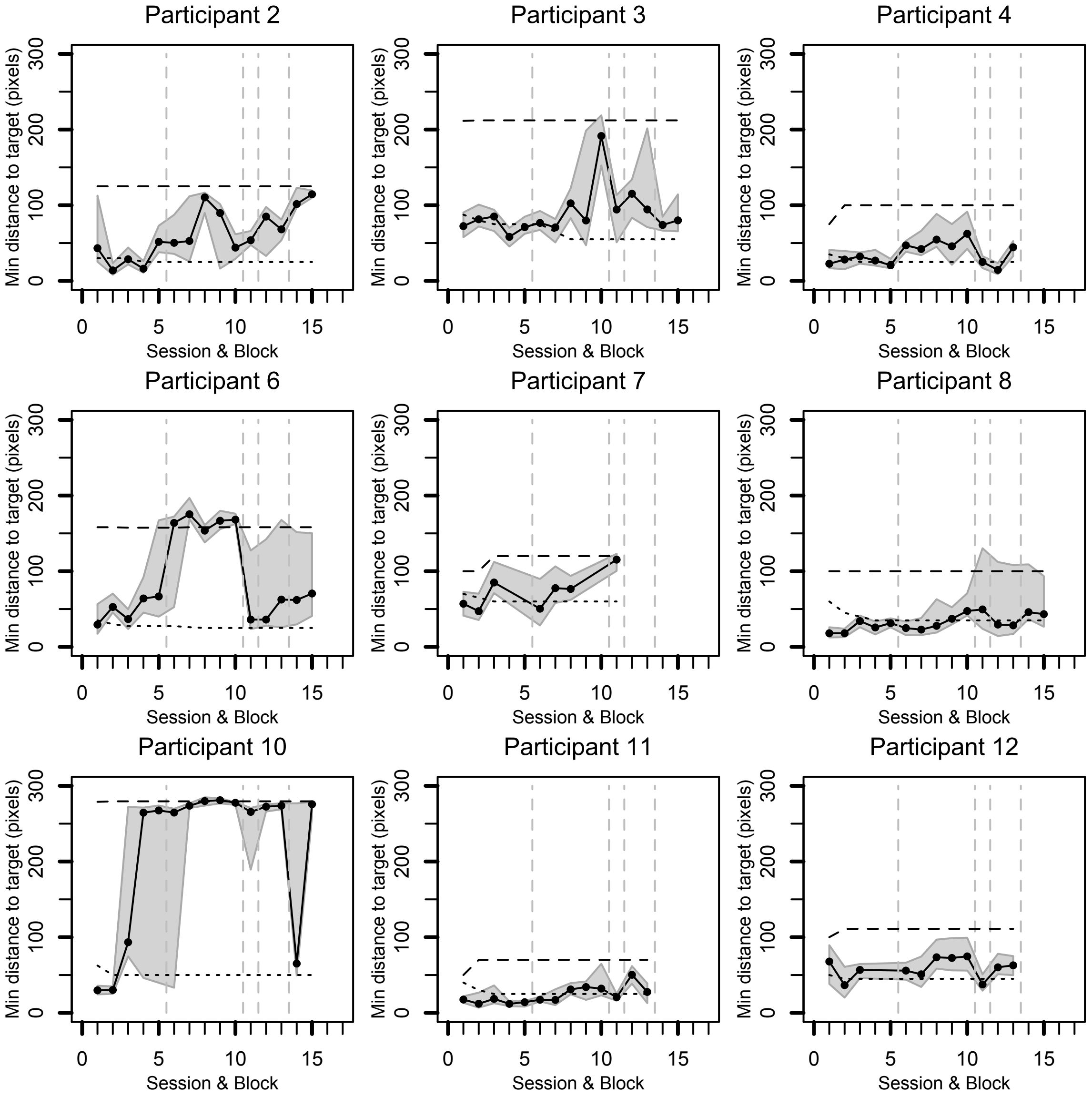

3. **Time since trial start plot for participants 2, 3, 4, 6, 7, 8, 10, 11, 12 (each in an individual plot). Layout is similar to the 2nd row of Figure 6 in the main manuscript.**

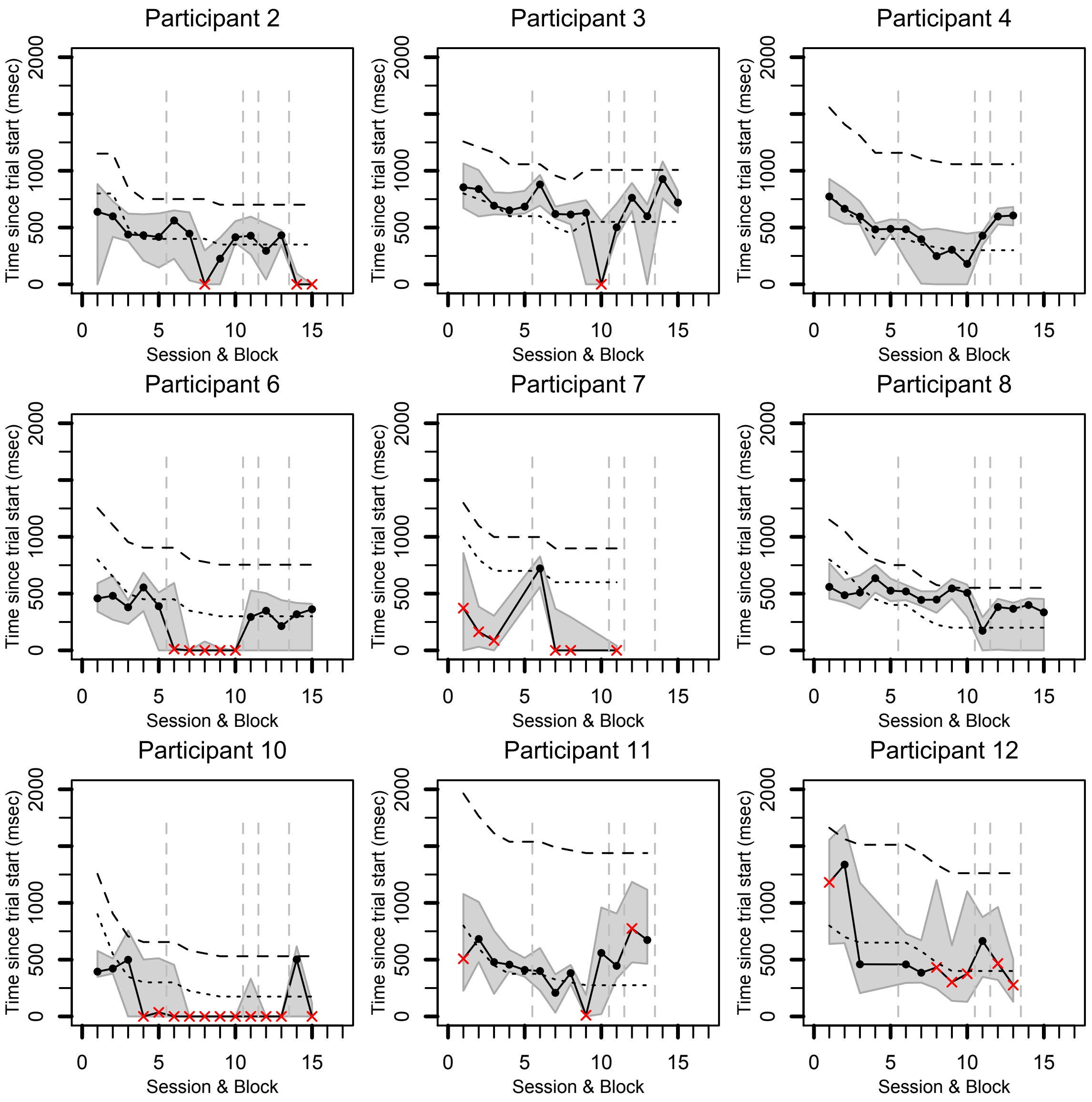

4. **Combined plot of time since trial start (vertical) and distance to target plot for participants 2, 3, 4, 6, 7, 8, 10, 11, 12 (each in an individual plot). Layout is similar to the 3rd row of Figure 6 in the main manuscript.**

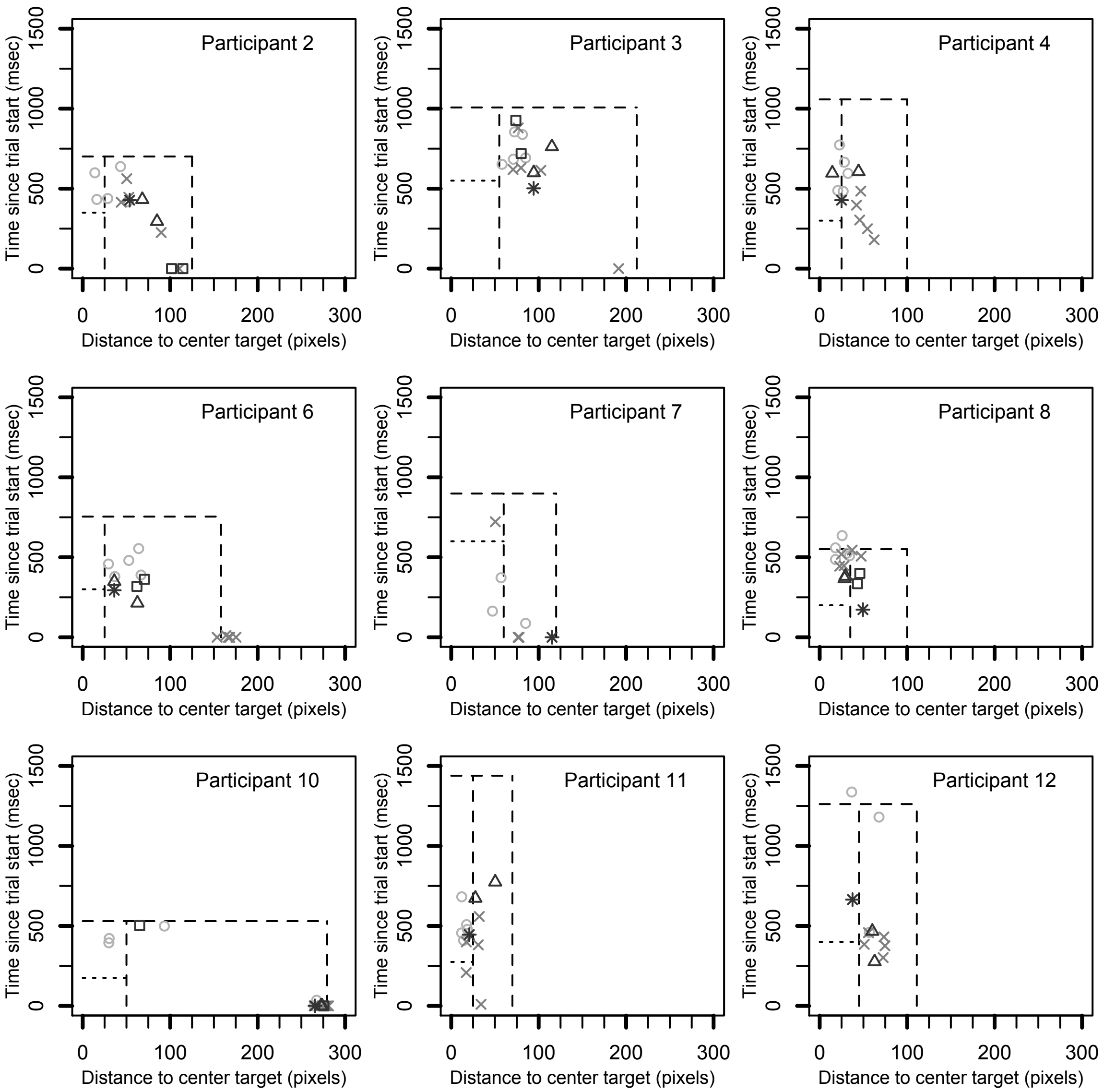

5. **Estimated performance plot of time and distance for participants 2, 3, 4, 6, 7, 8, 10, 11, 12 (each in an individual plot). Layout is similar to the 4th row of Figure 6 in the main manuscript.**

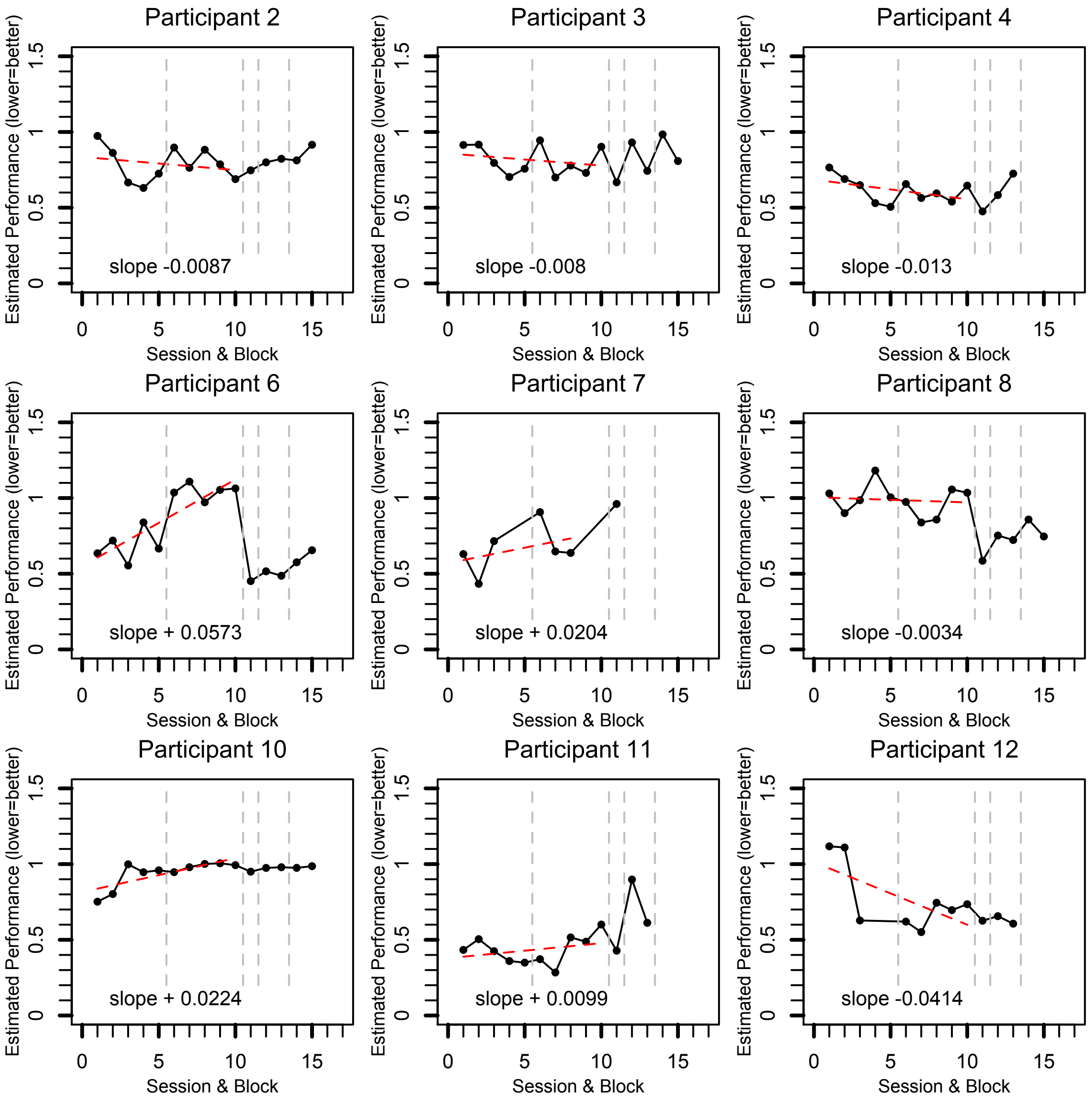

6. Proportion of saccades towards the scotoma in the visual search task for participants 2, 3, 4, 6, 7, 8, 10, 11, 12 (each in an individual plot). Horizontal axis plots the visit. (see text for detail)

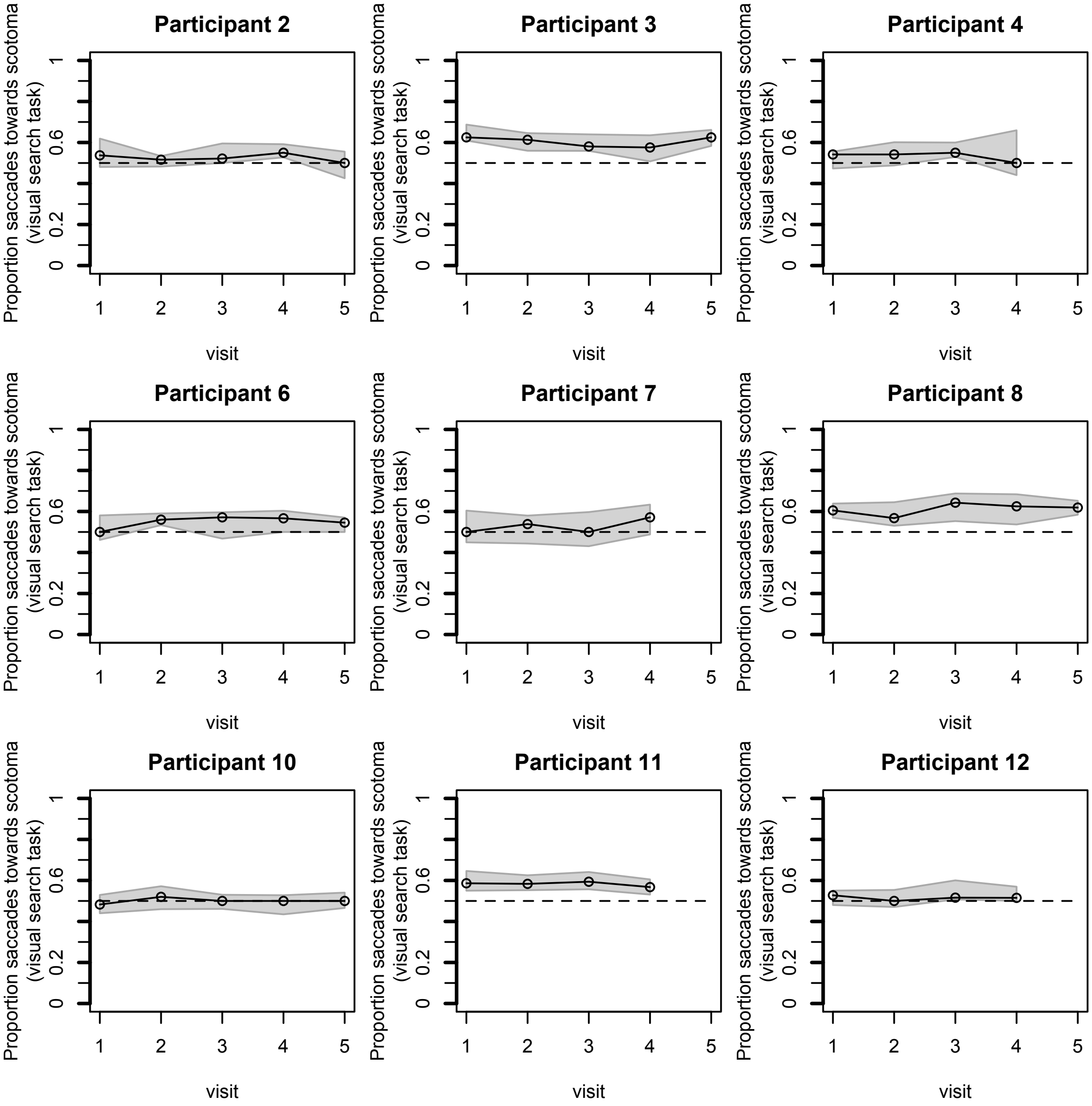

7. Subjective Questionnaire

The following are the questions we asked in the scotoma awareness questionnaire. This questionnaire was used during each visit to track progress of subjective experience. Participants were asked to elaborate on their answers and the initial question was used as a conversation opener. Specifically, the aspects that are printed without bold print were possible angles for follow-up. Many participants used the first two questions at their own discretion to elaborate on their medical history.
When needed, the questions were rephrased if participants did not understand the original phrasing.

A. Are you able to see any blind spots or detects in your field of vision?
--> Under what circumstances? (when, how frequent?)

B. Have you ever had any evidence or experiences that lead you to believe that you have defects in your field of vision?
--> Under what circumstances? (when, how frequent?)

C. We want to investigate how aware people are of the location of their scotoma and the impact on their sight. As you know, you have vision loss. Are you particularly aware of how this affects your vision? To be more specific, if you look straight ahead and don't move your eyes, what do you think would be hidden from you? For example, the top-left, or bottom-right, or right in the center?

D. Clock without seeing it: If you were to look at the center of a clock and keep looking there. Would there be any digits that you think you would not see?
---> Later this was also verified by performing the clock test

The following 3 questions were only asked during retention visits after training was completed

E. During your last visit I trained you to look at a particular area. Do you remember what area I trained you on?

F. Has the training had an impact on your lift? If so, how; can you give examples?

G. Have you become more aware of information that you might be missing in this area? When, how, and under what circumstances?
